# Identification and Validation of 7-lncRNA Signature of Epigenetic Disorders by Comprehensive Epigenetic Analysis

**DOI:** 10.1155/2022/5118444

**Published:** 2022-02-21

**Authors:** Peng Cao, Fan Li, Yajie Xiao, Shan Hu, Kangle Kong, Peng Han, Jiaqi Yue, Yu Deng, Zhikun Zhao, Dongfang Wu, Lu Zhang, Bo Zhao

**Affiliations:** ^1^Department of Thoracic Surgery, Tongji Hospital, Tongji Medical College, Huazhong University of Science and Technology, 1095 Jiefang Avenue, Qiaokou District, Wuhan City, Hubei Province, China 430030; ^2^YuceBio Technology Co., Ltd., 4th Floor, Phase I, Dabaihui Center, No. 2002, Shenyan Road, Haishan Street, Yantian District, Shenzhen City, Guangdong Province, China 518000; ^3^Department of Oncology, Tongji Hospital, Tongji Medical College, Huazhong University of Science and Technology, No. 1095 Jiefang Avenue, Wuhan, Hubei Province, China 430030

## Abstract

The survival rate of patients with lung adenocarcinoma (LUAD) is low. This study analyzed the correlation between the expression of long noncoding RNA (lncRNA) and epigenetic alterations along with the investigation of the prognostic value of these outcomes for LUAD. Differentially expressed lncRNAs were identified based on multiomic data and positively related genes using DESeq2 in R, differentially histone-modifying genes specific to LUAD based on histone modification data, gene enhancers from information collected from the FANTOM5 (Function Annotation Of The Mammalian Genome-5) (fantom.gsc.riken.jp/5) human enhancer database, gene promoters using the ChIPseeker and the human lincRNAs Transcripts database in R, and differentially methylated regions (DMRs) using Bumphunter in R. Overall survival was estimated by Kaplan-Meier, comparisons were performed among groups using log-rank tests to derive differences between sample subclasses, and epigenetic lncRNAs (epi-lncRNAs) potentially relevant to LUAD prognosis were identified. A total of seven dysregulated epi-lncRNAs in LUAD were identified by comparing histone modifications and alterations in histone methylation regions on lncRNA promoter and enhancer elements, including H3K4me2, H3K27me3, H3K4me1, H3K9me3, H4K20me1, H3K9ac, H3K79me2, H3K27ac, H3K4me3, and H3K36me3. Furthermore, 69 LUAD-specific dysregulated epi-lncRNAs were identified. Moreover, lncRNAs-based prognostic analysis of LUAD samples was performed and explored that seven of these lncRNAs, including A2M-AS1, AL161431.1, DDX11-AS1, FAM83A-AS1, MHENCR, MNX1-AS1, and NKILA (7-EpiLncRNA), showed the potential to serve as markers for LUAD prognosis. Additionally, patients having a high 7-EpiLncRNA score showed a generally more unfavorable prognosis compared with those which scored lower. Seven lncRNAs were identified as markers of prognosis in patients with LUAD. The outcomes of this research will help us understand epigenetically aberrant regulation of lncRNA expression in LUAD in a better way and have implications for research advances in the regulatory role of lncRNAs in LUAD.

## 1. Introduction

At present, lung cancer is the deadliest malignancy throughout the globe and is the leading cause of mortalities caused by cancer [1]. Lung adenocarcinoma (LUAD) composes 40% of all lung cancer incidence and is known as the most widely known histological subtype of lung cancer, with a high risk of developing distant metastasis during all stages [2]. Currently, many cases on the pathogenesis of LUAD have been reported [3–11]. For instance, N6-methyladenosine- (m6A-) related genes are expressed in LUAD and have a certain prognostic value. Furthermore, there has also been rapid clinical advancement of treatment strategies and personalized therapies for precision medicine, such as the improvement and application of tyrosine kinase inhibitors (TKIs), surgery, chemoradiotherapy, and immunotherapy [12]. Nevertheless, LUAD shows an overall survival (OS) rate of only around 16% for five years to date [13–16]. Additionally, only a small proportion of patients benefit from immunotherapy. Hence, discovering possible biomarkers for effective prognostic prediction is a crucial requirement.

Epigenetics includes heritable alterations in the chromatin that has the potential to alter gene expression without modifying the DNA coding sequence, including DNA methylation, microRNA regulation, and histone modifications [17, 18]. Information regarding different epigenetic events can help us understand the pathogenesis of cancer deeply and facilitate the discovery of prognostic biomarkers and new therapeutic targets. Histones act as the fundamental components of nucleosomes, which are the basic components of chromatin. Histone tails go through a process of extensive posttranslational modifications, such as acetylation and methylation [18]. According to research, gene mutations in histone modification regions are indicative of the prognosis of LUAD patients after surgery [19]. Moreover, it has been reported in another study certain histone lysine methyltransferases/demethylases, such as UTX and MLL4. EZH2 epigenetically regulate the expression of EMT-TFs, for example, Slug, Twist, and ZEB1. Hence, they may facilitate cancer metastasis from the lungs to the brain [20]. Dysregulation of these epigenomic regulatory processes has been found to be able to result in abnormal gene expression, therefore correlating with cancer risks and unfavorable clinical results.

Recent research has been conducted to create genetic markers predictive of the prognostic risk of LUAD patients. For instance, seven gene markers were discovered with the help of microarray data from the Gene Expression Omnibus (GEO) dataset (http://www.ncbi.nlm.nih.gov/geo) for early-stage LUAD [21]. In addition, 96 genes including five long noncoding RNAs (lncRNAs) that were related to prognosis in the test data using LUAD RNA sequencing and clinical data from The Cancer Genome Atlas (TCGA) (https://www.cancer.gov/about-nci/organization/ccg/research/structural-genomics/tcga) were discovered in the training data [22]. One study reported that the lncRNA expression models were used for the prediction of stage I LUAD and the construction of a 31-lncRNA signature to predict overall survival (OS) in LUAD patients [23]. Some studies have identified various genetic markers for prognostic risk prediction using a variety of methods [24–26]. Nevertheless, none of these studies had an epigenomic perspective on genetic markers for predicting LUAD prognostic risk.

Advances in multiomic technologies have promoted the application of different bioinformatics techniques and expression profiles, creating a better approach for prognostic assessment for patients with LUAD [22, 27, 28]. Nevertheless, these studies are missing a systematic identification of LUAD-related lncRNAs to date. The study of promoters and enhancers of lncRNAs at the epigenomic level can disclose the correlation between lncRNA expression and epigenetic alterations. The identification of regulatory pathways related to lncRNAs has prognostic importance for patients with LUAD and can improve their clinical outcomes.

## 2. Material and Methods

### 2.1. Expression Profile Data and Its Preprocessing

The gene expression profiles of LUAD and their corresponding normal samples, for instance, fragments per kilobase million (FPKM) and count along with clinical data, were acquired from TCGA database. Then, the FPKM was converted into TPM (transcripts per million). We obtained the GTF file from the GENCODE database (https://www.gencodegenes.org/human/), distinguished lncRNAs and protein coding genes (PCG) according to the gene types reported in GTF, and finally converted Ensembl IDs into gene symbols. Afterward, the expression profiles were divided into 59 normal samples and 513 LUAD samples on the basis of TCGA sample numbers (Supplementary Table 1). The work flow chart of this study is in Figure S1.

### 2.2. 450K Methylation Microarray Data and Its Preprocessing

TCGA database provided microarray data for LUAD. Subsequently, the microarray data were divided into 458 LUAD and 32 normal samples according to the sample numbers of LUAD. Missing values in LUAD data were filled by the *K*-nearest neighbor (KNN) method; particularly, the method identified neighboring points by distance measurement and estimated the missing values using the full value of neighboring observations. Specifically, remove the CpG sites with NA values exceeding 70% in all samples, remove the CpG sites with cross-reactivity in the genome according to the cross-reactive sites provided by Chen et al. [29], fill the missing values in the methylation spectrum using the KNN method of R-package impute [30], and further remove the unstable genomic methylation sites. That is, CPG sites and single nucleotide sites on sex chromosomes were removed.

### 2.3. Histone Data and Its Preprocessing

In the current report, the ENDCODE database provided replicated narrow peak data (hg38) of ten histone modifications along with H3K4me2, H3K27me3, H3K4me1, H3K9me3, H4K20me1, H3K9ac, H3K79me2, H3K27ac, H3K4me3, and H3K36me3, of the LUAD cell line PC-9 and the normal cell line IMR-90.

### 2.4. Identification of Dysregulated epi-lncRNAs and PCGs

Initially, the normalized gene expression profile count provided by TCGA was converted into an integer-type count, and then, the differentially expressed PCGs and lncRNAs were identified with DESeq2 (https://www.bioconductor.org/packages/release/bioc/html/DESeq2) [31] in R; *P* values were determined by the Benjamini-Hochberg method, where lncRNAs or PCGs showing a false discovery rate (FDR) < 0.05 were deemed as a significance. Subsequently, according to the physical location of the histone modification peaks, this study identified that LUAD-specific peaks and differential peaks were retained when peaks have a *P* value < 0.05. Furthermore, the differential peaks were combined with the gene transfer format file in the GENCODE database to collect genes with differential histone modifications and identify gene enhancers from the data available in the FANTOM5 human enhancer database. With regions of gene promoters generally defined as 2 kb upstream and 0.5 kb downstream of the transcription start site, promoters of genes were identified with the help of ChIPseeker [32] in R and the human lincRNAs Transcripts database (http://t.cn/zW2uZyY). Moreover, the Bumphunter method of CHAMP [33] in R identified the differentially methylated regions (DMRs), where regions with a BumphunterDMR.*P* value < 0.001 were deemed as significant DMRs. Lastly, this study defined the dysregulated epi-lncRNAs and PCGs following the stated criteria: (1) differentially expressed lncRNAs and PCGs in LUAD tissue compared to the normal tissue samples and (2) promoters or enhancers showing overlap in at least one differentially histone modified or differentially methylated region (called non-epi-PCG, non-epi-lncRNA, epi-lncRNA, and epi-PCG).

### 2.5. The Genomic Signal Characterization of Dysregulated epi-lncRNAs

Four classes of mRNA, including non-epi-lncRNA, non-epi-PCG, epi-PCG, and epi-lncRNA, were characterized by the length and number of exons, transcripts, and genes.

### 2.6. The Genomic Mapping of Dysregulated epi-lncRNAs Showing Histone Modifications

Analysis of the distribution features of enhancers and promoters of epi-lncRNAs with various histone modifications on the genome was carried out.

### 2.7. Recognition of Lung Adenocarcinoma-Specific Candidate Dysregulated epi-lncRNAs

Lnc2Cancer (version: 3.0) (http://bio-bigdata.hrbmu.edu.cn/lnc2cancer) helped in the analysis of epi-lncRNAs that overlapped with already identified cancer lncRNAs. To investigate the regulation of epi-lncRNAs in LUAD further, the LUAD-associated epi-lncRNAs were screened. Additionally, taking into consideration that most genes affecting the disease progression exhibit dysregulated expression, the TPM expression profile data acquired from TCGA was used for the identification of epi-lncRNAs with substantially altered expression in LUAD.

### 2.8. Survival Model Construction and Validation of Dysregulated epi-lncRNAs

As per the abovementioned epi-lncRNA expression values, samples were sorted into two groups of high and low expression. Afterward, sample analysis was carried out for OS and survival status according to different sample categories. The Kaplan-Meier method estimated survival rates and the time of survival. In particular, all samples were listed lengthwise during the follow-up period. Afterward, the survival curve showing time on the horizontal axis and survival rate on the vertical axis was plotted by the Kaplan-Meier method, which portrays the features of patient survival over time and makes full use of the incomplete data provided by the censored data in the measurement of the survival rate. A survival model was created considering that no endpoint events happened in patients during the follow-up time. Group comparisons were carried out using log-rank tests to derive differences between sample subclasses and for the identification of epi-lncRNAs potentially relevant to the prognosis of LUAD patients.

## 3. Results

### 3.1. Identification and Genomic Characterization of Dysregulated epi-lncRNAs and PCGs

**Figure 1:**
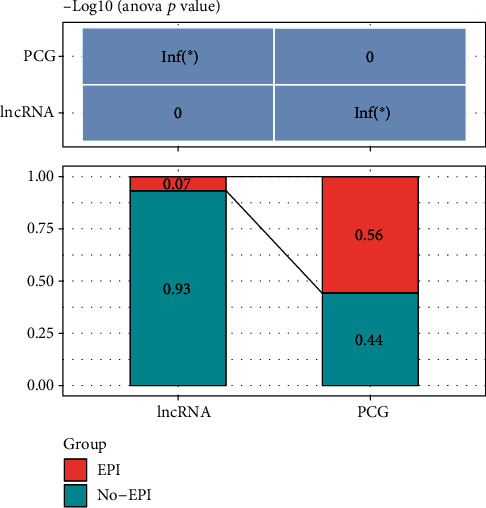
epi-lncRNAs and epi-PCGs as a percentage of all lncRNAs and PCGs on the genome, respectively.

For the investigation of the potential regulatory correlation between lncRNA expression and epigenetic alterations in LUAD, DESeq2 helped in the identification of substantially differentially expressed lncRNAs and genes containing 13,897 PCGs and 6,206 lncRNAs, respectively. Furthermore, combining 450K methylation microarray data and histone modification data, 906 epi-lncRNAs, 10,793 epi-PCGs, 8,543 non-epi-PCGs, and 12,424 non-epi-lncRNAs were identified, showing that lncRNAs exhibited a lower aberration frequency in LUAD than PCGs (Figure 1).

**Figure 2:**
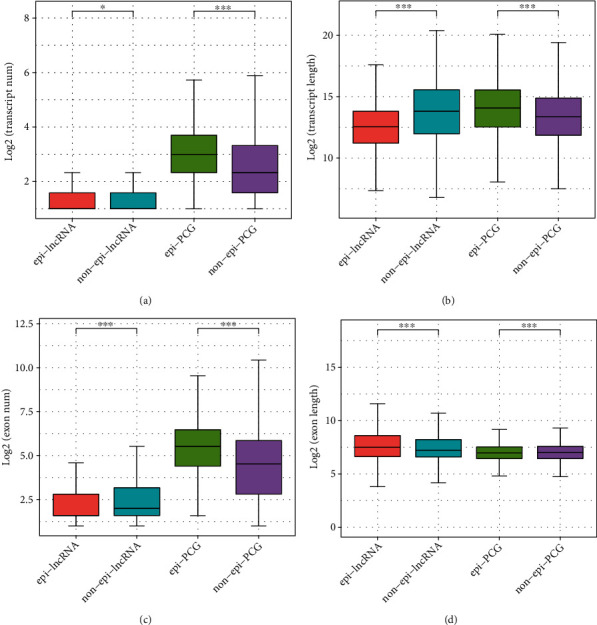
Comparison of genomic features between dysregulated epi-lncRNAs/PCGs and non-epi-lncRNAs/PCGs: (a) comparison of transcript numbers; (b) comparison of transcript lengths; (c) comparison of exon numbers; (d) comparison of exon lengths.

This report further compared the number and length of gene exons and transcripts of epi-lncRNAs, non-epi-lncRNAs, epi-PCGs, and non-epi-PCGs to describe the genomic profile of dysregulated epi-lncRNAs. The epi-lncRNAs had smaller transcripts as compared to non-epi-lncRNAs. The transcript lengths of epi-lncRNAs were smaller in comparison to those of non-epi-lncRNAs. In comparison to non-epi-PCG ones, epi-PCG transcripts were more in number and were lengthier (Figures 2(a) and 2(b)). Additionally, epi-lncRNAs had fewer exons, but they were longer as compared to the non-epi-lncRNAs, while epi-PCG had more exons in comparison with non-epi-PCG (Figures 2(c) and 2(d)).

**Figure 3:**
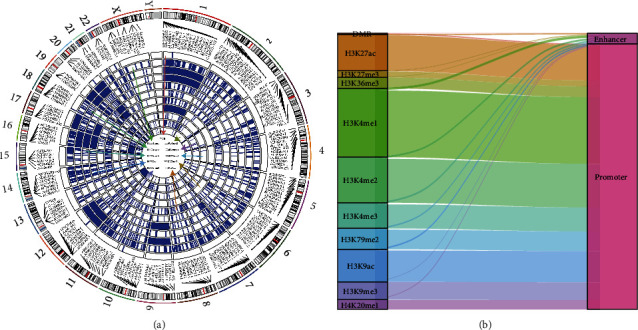
Distribution of epi-lncRNAs with a variety of histone modifications on the genome: (a) genomic landscape of epi-lncRNAs with different histone modifications and differential regions; (b) distribution of epi-lncRNA types.

A systematic analysis of epi-lncRNAs in LUAD was done, highlighting the genomic landscape of epi-lncRNAs with various DMRs and histone modifications. As illustrated in Figure 3(a), the aberrant histone modifications in these lncRNAs mainly included H3K4me3, H4K20me1, H3K4me2, H3K4me1, H3K9ac, H3K27ac, H3K20me1, and H3K79me2. Most of the epi-lncRNAs were together with multiple aberrant histone modifications, which were largely concentrated in the promoter region (Figure 3(b)).

### 3.2. Potential Functions of Dysregulated epi-lncRNAs

**Figure 4:**
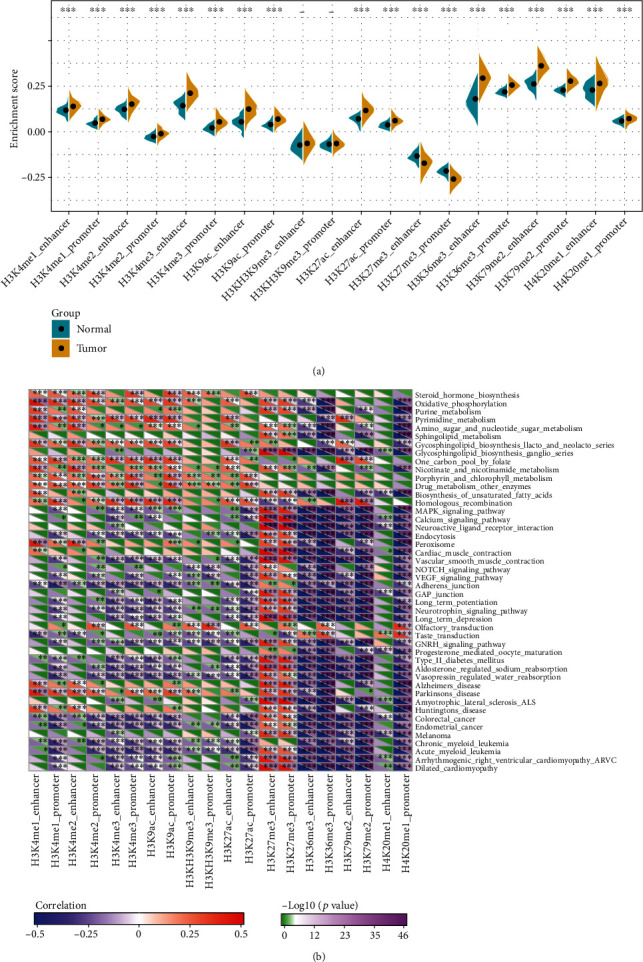
Functional analysis of epi-lncRNAs: (a) differences of 20 dysregulated epi-lncRNAs among cancer samples and paracancerous samples; (b) most relevant KEGG pathway for 20 dysregulated epi-lncRNAs.

For the purpose of illustrating the potential functions of these dysregulated epi-lncRNAs formed as a result of histone modifications, a systematic analysis between the expressions of epi-lncRNAs and the pathways in LUAD was carried out. In particular, the expression profiles of lncRNAs with a variety of histone modifications were extracted separately, and the enrichment scores of these lncRNAs were measured for individual samples with the help of single-sample gene set enrichment analysis (ssGSEA). H3K4me1_enhancer, H3K4me1_promoter, H3K4me2_enhancer, H3K4me2_promoter, H3K4me3_enhancer, H3K4me3_promoter, H3K9ac_enhancer, H3K9ac_promoter, H3K27ac_enhancer, H3K27ac_promoter, H3K36me3_enhancer, H3K36me3_promoter, H3K79me2_enhancer, H3K79me2_promoter, H4K20me1_enhancer, and H4K20me2_promoter had substantially enhanced enrichment scores in the tumor samples in comparison to those in the paracancerous samples, while H3K27me3_enhancer and H3K27me3_promoter had greatly increased enrichment scores in the paracancerous samples as compared to those in the tumor samples (Figure 4(a)). These results indicated that H3K27me3_enhancer and H3K27me3_promoter may have a protective effect, while H3K4me1_enhancer, H3K4me1_promoter, H3K4me2_enhancer, H3K4me2_promoter, H3K4me3_enhancer, H3K4me3_promoter, H3K9ac_enhancer, H3K9ac_promoter, H3K27ac_enhancer, H3K27ac_promoter, H3K36me3_enhancer, H3K36me3_promoter, H3K79me2_enhancer, H3K79me2_promoter, H4K20me1_enhancer, and H4K20me2_promoter may have procarcinogenic effects.

Moreover, the Kyoto Encyclopedia of Genes and Genomes (KEGG) pathway score of the samples in this study was evaluated; moreover, we assessed that the correlation between the KEGG pathway and the enrichment score of each epi-lncRNA was observed for the purpose of deriving the linked KEGG pathway of each epi-lncRNA. These findings demonstrated that there were 46 KEGG pathways most related to the 20 epi-lncRNAs (Figure 4(b)), showing that there was a certain amount of consistency in the various types of epi-lncRNA-associated pathways. These 46 pathways included the tumor-associated COLORECTAL_CANCER and ENDOMETRIAL_CANCER, along with the metabolism-associated NICOTINATE_AND_NICOTINAMIDE_METABOLISM, AMINO_SUGAR_AND_NUCLEOTIDE_SUGAR_METABOLISM, and PYRIMIDINE_METABOLISM. Generally, these outcomes highlighted that epi-lncRNAs were closely linked to tumorigenesis, tumor progression, and metabolism.

### 3.3. Relationship between Dysregulated epi-lncRNAs and RNA Modifications

**Figure 5:**
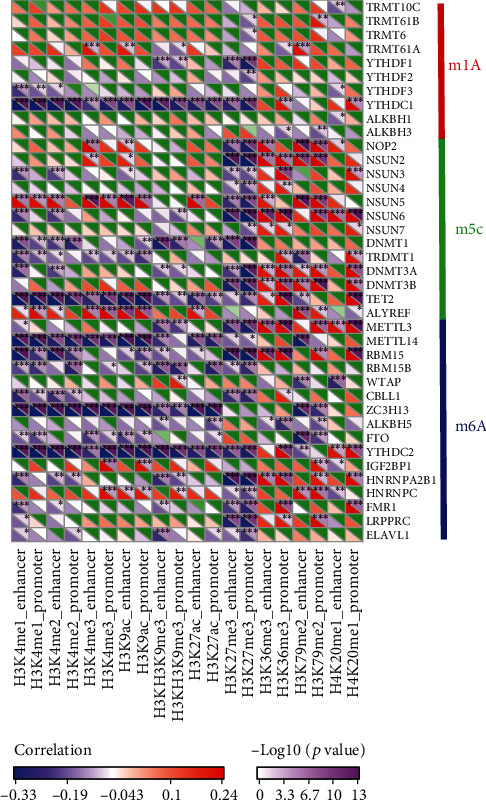
Correlation of enrichment scores of 20 histone modifications with m6A, m5C, and m1A genes.

RNA modifications are significant epigenetic features that are linked to various significant biological processes. This report analyzed the correlation between 20 different histone modifications and the m6A and m5C genes. Particularly, the gene expression profiles of m6A, m5C, and m1A were extracted from TCGA LUAD expression profiles, and the relation between the enrichment scores of 20 histones and the m6A, m5C, and m1A genes was measured (Figure 5). Additionally, these enrichment scores were greatly linked to the m6A, m5C, and m1A genes. Furthermore, H3K4me2, H3K4me1, H3K36me3, H3K9ac, H3K4me3, H3K27ac, H3K9me3, H3K27me3, H4K20me1, and H3K79me2 had both common and specific relations with the aforementioned three genes, indicating a possible presence of various modes of regulation of lncRNA dysregulation as a result of histone modification in promoter and enhancer. Generally, those epi-lncRNAs have a close link to RNA modifications.

### 3.4. Correlation between LUAD-Specific Dysregulated epi-lncRNAs with Prognosis

**Figure 6:**
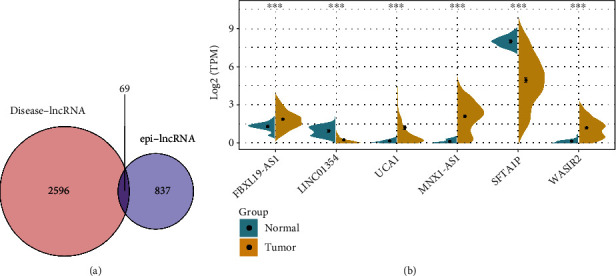
Analysis of epi-lncRNAs specific to LUAD: (a) intersection of disease-associated lncRNAs with LUAD-associated epi-lncRNAs; (b) differential expression of LUAD-associated epi-lncRNAs in tumor and normal samples.

Outcomes of a comparative analysis of the 2665 disease-associated lncRNAs provided by the Lnc2Cancer database (version: 3.0) showed that 69 of the 906 epi-lncRNAs identified were lncRNAs linked to known human cancers (Figure 6(a)). To better understand the regulatory role of candidate lncRNAs in LUAD, a collection of LUAD-associated lncRNA genes were obtained in this study from the Lnc2Cancer database (version: 3.0), and it was observed that six out of the 69 epi-lncRNAs have been reported to have a direct link to LUAD. The differential expression of each of the six epi-lncRNAs in LUAD samples and normal samples was then calculated (Figure 6(b)), and the outcomes highlighted that all six epi-lncRNAs were greatly differentially expressed in both the normal and tumor samples: FBXL19-AS1, UCA1, MNX1-AS1, and WASIR2 were expressed more in tumor tissues as compared to those in the paracancerous tissues, while the lncRNAs LINC01354 and SFTA1P were expressed more in paracancerous tissues in comparison with tumor tissues. All of the six lncRNAs had histone modifications in the promoter region. Particularly, the promoter region of FBXL19-AS1 had histone H3K9me3 and H3K79me2 modifications; the promoter region of LINC01354 had histone H3K4me1 and H3K4me3 modifications; the promoter region of UCA1 had H3K4me1, H3K9ac, H3K79me2, and H3K36me3 modifications; the promoter region of MNX1-AS1 had histone H3K4me1, H3K4me2, and H3K9ac modifications; the promoter region of SFTA1P had histone H3K4me3, H3K4me2, H3K4me1, H3K9me3, H3K36me3, H3K79me2, and H3K9ac modifications; the promoter region of WASI R2 had H3K4me2 modification.

Specifically, the FBXL19-AS1 and MNX1-AS1 were observed to be upregulated in the Lnc2Cancer database (version: 3.0), while the SFTA1P was downregulated in the Lnc2Cancer database (version: 3.0); LINC01354, UCA1. Also, the WASIR2 was shown to be differentially expressed in the Lnc2Cancer database (version: 3.0). On the contrary, the expression of FBXL19-AS1, UCA1, MNX1-AS1, and WASIR2 was upregulated in TCGA database, while the LINC01354 and SFTA1P were downregulated in TCGA database. Overall, six lncRNAs (50%) expressed in TCGA dataset were similar to the Lnc2Cancer database (version: 3.0).

**Figure 7:**
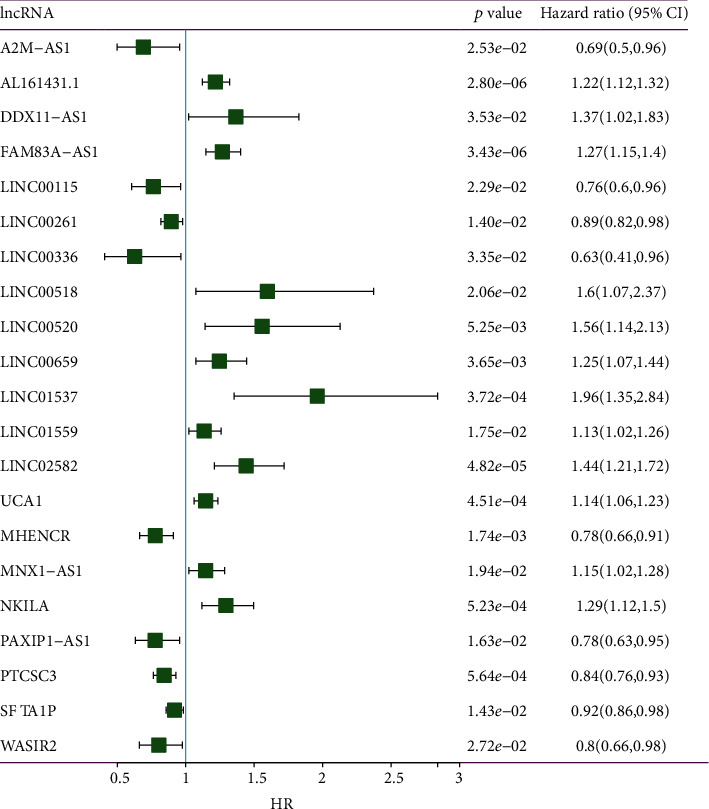
Forest plot of univariate analysis of 21 LUAD-associated epi-lncRNA genes.

For a deeper understanding of the potential prognostic significance of dysregulated epi-lncRNA, survival analysis was performed on LUAD samples from TCGA using 69 epi-lncRNAs from the abovementioned outcomes. A univariate Cox analysis found that 21 lncRNAs were linked to survival, including A2M-AS1, AL161431.1, DDX11-AS1, FAM83A-AS1, LINC00115, LINC00261, LINC00336, LINC00518, LINC00520, LINC00659, LINC01537, LINC01559, LINC02582, UCA1, MHENCR, MNX1-AS1, NKILA, PAXIP1-AS1, PTCSC3, SFTA1P, and WASIR2 (Figure 7). Furthermore, using the max_stat function in R to truncate these 21 lncRNAs separately to divide the high and low expression groups, it was observed that except for the nonsignificant survival curves of LINC01537 and LINC02582, the survival curves of the other 19 lncRNAs were significant (Figure S2).

**Figure 8:**
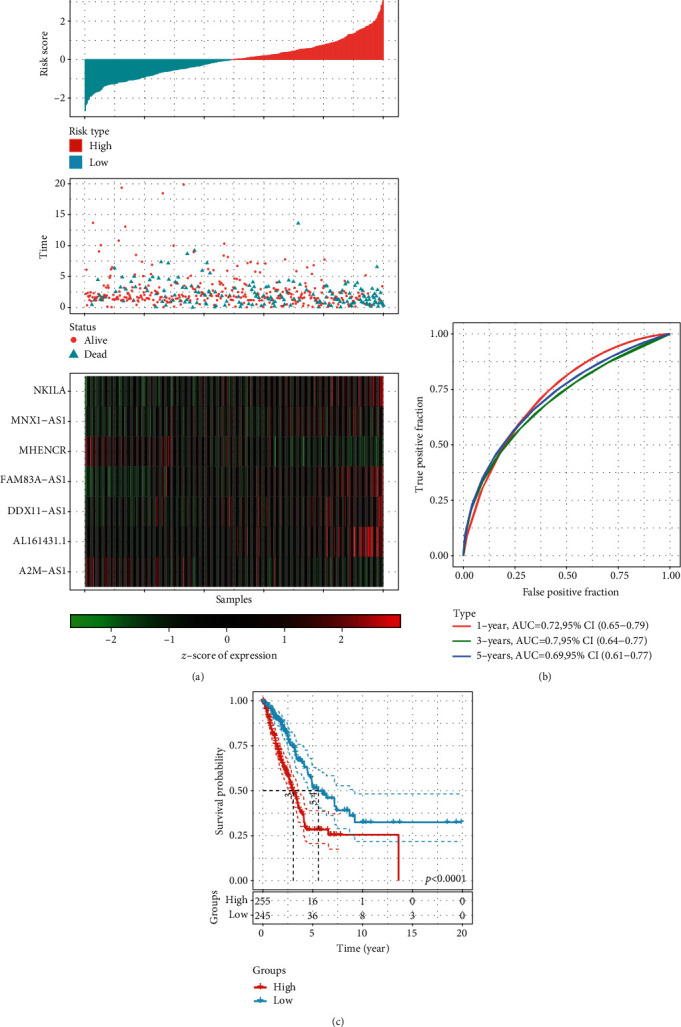
Prognostic analysis of seven LUAD-specific lncRNAs: (a) heat map analysis of 7-EpiLncRNA scores with patient survival status and with A2M-AS1, AL161431.1, DDX11-AS1, FAM83A-AS1, MHENCR, MNX1-AS1, and NKILA in TCGA dataset, where red represents the high-risk group and blue represents the low-risk group; (b) ROC analysis of the 7-EpiLncRNA score model in TCGA dataset; (c) Kaplan-Meier survival analysis of the high-risk and low-risk groups in TCGA dataset.

In order to construct the prognostic model of the dysregulated epi-lncRNAs, the 69 LUAD-associated epi-lncRNA genes were reduced to 21 based on a univariate Cox analysis, 19 out of which were screened for study by survival analysis. The stepwise regression method helped in the further decreasing of the number of genes, which utilizes the Akaike info criterion (AIC) and considers statistical fit of a model and parameter numbers. The stepAIC method in the MASS package (https://www.rdocumentation.org/packages/MASS) begins with the most complex model and then removes one variable to decrease the AIC; a smaller value indicated a better model, showing that the model has gained a sufficient fit with fewer parameters. With the help of this algorithm, this study eventually reduced the 19 epi-lncRNAs to seven as prognostic markers, including A2M-AS1, AL161431.1, DDX11-AS1, FAM83A-AS1, MHENCR, MNX1-AS1, and NKILA. Multivariate survival analysis in TCGA dataset was used to construct a 7-EpiLncRNA model, with higher 7-EpiLncRNA scores associated with a higher death rate (Figure 8(a)), and lower 7-EpiLncRNA scores indicated reduced mortality. Among the seven lncRNAs, MHENCR and A2M-AS1 had similar expression patterns with each other. However, the other five lncRNAs had more similar expression patterns with each other. Consequently, the seven lncRNAs were sorted into two groups. Moreover, the receiver operating characteristic (ROC) curve analysis indicated that the 7-EpiLncRNA score had one-, three-, and five-year AUC values of 0.72, 0.7, and 0.69, respectively, showing a good prognosis (Figure 8(b)). Patients with a high 7-EpiLncRNA score had a substantially worse prognosis as compared to those with a low 7-EpiLncRNA score (Figure 8(c)). We compared the relationship between T.stage, N.stage, M.stage, stage, gender, age, and 7-EpiLncRNA. Univariate analysis showed that the characteristics of T.stage, N.stage, M.stage, stage, and 7-EpiLncRNA were significantly correlated with poor prognosis (Figure S3A). Multivariate survival analysis showed that the characteristics of T.stage, N.stage, and 7-EpiLncRNA were significantly correlated with poor prognosis, of which 7-EpiLncRNA was the most significant (Figure S3B), These results suggest that 7-EpiLncRNA is an independent prognostic factor. In order to evaluate the relationship between 7-EpiLncRNA and immunity, we first evaluated the immune infiltration score of each patient by using R software package Estimate [34] and compared the difference of immune infiltration in patients in the high-risk and low-risk groups. It can be observed that high-risk patients have lower immune infiltration (Figure S3C). Further, we evaluated the relationship between immune cell infiltration in 22 of each patient by using R software package ciberport [35]. By analyzing the difference of infiltration in these immune cells in patients of the high-risk and low-risk groups, it was observed that T cells CD4 memory resting, dendritic cells resting, and mast cells resting were significantly higher in low-risk patients, and T cells CD4 memory activated and macrophages M0 were significantly higher in tumor samples (Figure S3D). These results suggest that 7-EpiLncRNA has an important relationship with tumor immunity.

**Figure 9:**
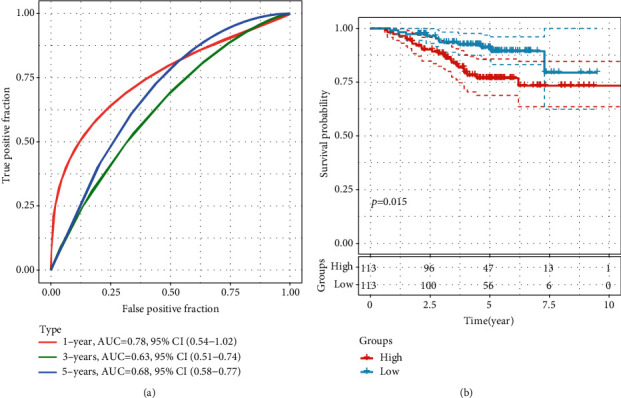
Validation of prognostic analysis of seven LUAD-specific lncRNAs based on GEO data: (a) ROC analysis of 7-EpiLncRNA score in the GSE31210 dataset; (b) prognostic differences among patients with high and low 7-EpiLncRNA scores in the GSE31210 dataset.

For the validation of the robustness of the 7-EpiLncRNA score model, the dataset GSE31210 with prognostic information was acquired by the GEO database (Supplementary Table 2), from which the expression profiles of seven epi-lncRNAs were collected. Additionally, the 7-EpiLncRNA score of each sample was determined by following the same procedure. From ROC analysis, it could be found that the 7-EpiLncRNA score had one-, three-, and five-year AUC values of 0.72, 0.7, and 0.69, respectively, with a good prognosis (Figure 9(a)). Patients with a high 7-EpiLncRNA score had a substantially worse prognosis as compared to those with a low 7-EpiLncRNA score (Figure 9(b)), which was supported by the previous outcomes.

## 4. Discussion

According to the multiomic data, many dysregulated epi-lncRNAs in LUAD were identified in this study by comparing the epigenetic modifications on lncRNA enhancer and promoter elements. Comparative analysis of the length and number of gene exons and transcripts of non-epi-lncRNAs, non-epi-PCGs, epi-PCGs, and epi-lncRNAs showed that the transcripts of epi-lncRNAs were smaller in length as compared to those of non-epi-lncRNAs, and the epi-PCG transcripts were more numerous and longer in length as compared to those of non-epi-PCGs (Figures 2(a) and 2(b)). These results may indicate that dysregulated epi-PCGs are few in genome distributions. Moreover, the smaller transcript lengths of epi-lncRNAs may be due to early transcript termination owing to the lncRNA dysregulation. In comparison with non-epi-lncRNAs, the epi-lncRNAs have fewer exons but are longer in length, while epi-PCGs have more exons as compared to non-epi-PCGs. The higher number of exons also indicated the complexity of epi-PCG in the regulation of alternative splicing. Additionally, lncRNA dysregulation caused by aberrant histone modifications showed that H3K27me3 may have a protective role, while other histone modifications may be involved in prooncogenic activities. This may be because the modifications of H3K27me3 in the promoter and enhancer regions inhibit the transcriptional initiation and enhancement of these cancer-associated dysregulated epi-lncRNAs or genes, thereby making them less functional or inactive. Other histones, for instance, acetylation, improve the transcriptional initiation and activity of cancer-associated dysregulated epi-lncRNAs or genes, thus making them more functional and facilitating the further progression of cancer.

Afterward, LUAD-specific dysregulated epi-lncRNAs were identified in this study, and we performed the prognostic analysis. Seven of these lncRNAs, including A2M-AS1, AL161431.1, DDX11-AS1, FAM83A-AS1, MHENCR, MNX1-AS1, and NKILA, were shown to be potential candidate target markers. The results of several reported studies support the findings of this research. For instance, A2M-AS1 has been reported to have regulatory effects on downstream factors such as CD2 and SELL, in the cell adhesion molecule pathway, indicating that A2M-AS1 may be a visible candidate prognostic factor and therapeutic target for breast cancer [36]. Research also suggests that the high expression levels of AL161431.1 are observed in endometrial cancer (EC) tissues and cells contained in the cytoplasm, and loss-of-function assays confirmed the oncogenic role of AL161431.1. It was noted through the established ceRNA network that AL161431.1, miR-1252-5p, and mitogen-activated protein kinase signaling pathways have a role in EC [37]. In melanoma tissues, MHENCR shows an upregulated expression and is further upregulated in metastatic melanoma, from which researchers indicated that an elevated MHENCR expression is related to a lower melanoma survival [38]. Studies, therefore, support that these three lncRNAs have regulatory functions in LUAD.

Additionally, in non-small-cell lung cancer (NSCLC) tumor tissues and cells, DDX11-AS1 is found to be upregulated. DDX11-AS1 knockdown has a substantial inhibitory effect on cell proliferation *in vitro* as well as *in vivo*. It was highlighted that DDX11-AS1 promotes NSCLC progression through the activation of the phosphatidylinositol 3 kinase-protein kinase B (PI3K/AKT) signaling pathway [39]. lncRNA FAM83A AS1 promotes LUAD progression by enhancing the expression levels of FAM83A [40]. NKILA expression shows a great downregulation in lung cancer tissues than the corresponding normal tissues. The low expression levels of lncRNA NKILA facilitate the progression of NSCLC, and this role is dependent on IL-11/STAT3 signal transduction [41]. The expression of NKILA in NSCLC tissues is downregulated than adjacent noncancerous tissues, and low-expressed NKILA in tumor tissues is closely related to the advanced tumor-node-metastasis (TNM) stage and lymph node metastasis [42]. The expression of MNX1-AS1 is greatly increased in lung cancer tissues in comparison with the normal lung tissues. High expression of MNX1-AS1 is linked to poor prognosis in NSCLC. Knockdown of MNX1-AS1 inhibits the migration, invasion, and proliferation of NSCLC cell line A549 and promotes apoptosis [43]. MNX1-AS1 is intensely upregulated in lung cancer, which was observed to be contained in the cytoplasm and interact with miR527. By inhibiting the availability of miR527 and MNX1-AS1, the expression of BRF2 is promoted. Restoration of BRF2 can reduce the defects in migration, invasion, and proliferation caused by MNX1-AS1 knockdown [44]. Also, it has been observed that high-expressed MNX1-AS1 has been found to be related to poor differentiation, tumor size, advanced clinical stage, distant metastasis, and lymph node metastasis of LUAD patients. Previous *in vitro* functional studies demonstrated that the inhibition of MNX1-AS1 suppresses LUAD cell migration and proliferation and also stimulates cell apoptosis [45]. The relevance of these four lncRNAs to LUAD and lung cancer described above further validates the results of this study.

The approach of multiomics-based analysis used in this research serves as a reference for other studies on different cancer types by clarifying the epigenetic modifications of promoters and enhancers on the genome for the analysis of genes or lncRNAs. Out of the seven lncRNAs identified, the upregulation of DDX11-AS1, AL161431.1, MNX1-AS1, and MHENCR may have a negative correlation with OS, showing an unfavorable prognosis of lung cancer patients. These four lncRNAs may have the potential to promote the progression of LUAD through the regulation of migration, proliferation, and invasion. Nevertheless, low expression of A2M-AS1 and NKILA may have a function in the progression of LUAD. Further experimental evidence is required even after the identification of marker lncRNAs as potential targets in this report, for the validation of the results of this study and to further investigate their possible epigenetic regulatory mechanisms.

## 5. Conclusions

Overall, 906 epi-lncRNAs, 12,424 non-epi-lncRNAs, 10,793 epi-PCGs, and 8,543 non-epi-PCGs were recognized on the basis of a multiomic dataset. lncRNAs were noted to have a much lesser frequency of aberrations in LUAD than PCGs, and 69 epi-lncRNAs were enriched in lncRNAs known to be correlated with a variety of human cancers. The epi-lncRNAs had longer exons that were more in number along with the transcripts. Dysregulated epi-lncRNAs had more exons, lengthier genes, and more transcripts as compared to non-epi-lncRNAs. Lastly, this study screened out seven LUAD-specific epi-lncRNAs and constructed a prognostic model according to them.

## Data Availability

The dataset used in this study could be publically found at GSE31210, in https://www.ncbi.nlm.nih.gov/geo/query/acc.cgi?acc=GSE31210.

## Supplementary Materials

Supplementary Materials Figure S1: work flow chart. Figure S2: the 21 lncRNA expression values were, respectively, used as truncation and divided into the high expression group and the low expression group, and the KM survival curves were calculated. Figure S3: relationship between 7-EpiLncRNA scores and clinical features and immunity: (A) univariate analysis of T.stage, N.stage, M.stage, stage, gender, age, and 7-EpiLncRNA forest map; (B) multivariate analysis of T.stage, N.stage, M.stage, stage, gender, age, and 7-EpiLncRNA forest map; (C) differences in immune infiltration scores between the high-risk and low-risk groups; (D) differences in 22 kinds of immune cell infiltration scores between the high- and low-risk groups. Supplementary Table 1: clinical characteristics of each sample in TCGA cohort. Supplementary Table 2: clinical characteristics of each sample in the GSE31210 cohort.
