# Supplementary material for: Identification and Validation of 7-lncRNA Signature of Epigenetic Disorders by Comprehensive Epigenetic Analysis
Source: Dis Markers. 2022 Feb 21;2022:5118444. doi: 10.1155/2022/5118444 (PMC8885251; doi:10.1155/2022/5118444)
Supplement: Supplementary Materials — Figure S1: work flow chart. Figure S2: the 21 lncRNA expression values were, respectively, used as truncation and divided into the high expression group and the low expression group, and the KM survival curves were calculated. Figure S3: relationship between 7-EpiLncRNA scores and clinical features and immunity: (A) univariate analysis of T.stage, N.stage, M.stage, stage, gender, age, and 7-EpiLncRNA forest map; (B) multivariate analysis of T.stage, N.stage, M.stage, stage, gender, age, and 7-EpiLncRNA forest map; (C) differences in immune infiltration scores between the high-risk and low-risk groups; (D) differences in 22 kinds of immune cell infiltration scores between the high- and low-risk groups. Supplementary Table 1: clinical characteristics of each sample in TCGA cohort. Supplementary Table 2: clinical characteristics of each sample in the GSE31210 cohort. [file 5118444.f1.pdf]

Supplementary materials legends

Figure S1 Work flow chart

Figure S2 The 21 lncRNA expression values were respectively used as truncation and divided into the high expression group and the low expression group, and the KM survival curves was calculated.

Figure S3 Relationship between 7-EpiLncRNA scores and clinical features and immunity. A: Univariate analysis of T.stage, N.stage, M.stage, Stage, Gender, age and 7-EpiLncRNA forest map. B: Multivariate analysis of T.stage, N.stage, M.stage, Stage, Gender, age and 7-EpiLncRNA forest map. C: Differences in immune infiltration scores between high-risk and low-risk groups. D: Differences in 22 kinds of immune cell infiltration scores between high and low risk groups.

Supplementary Table1: Clinical characteristics of each sample in TCGA cohort

Supplementary Table2: Clinical characteristics of each sample in GSE31210 cohort

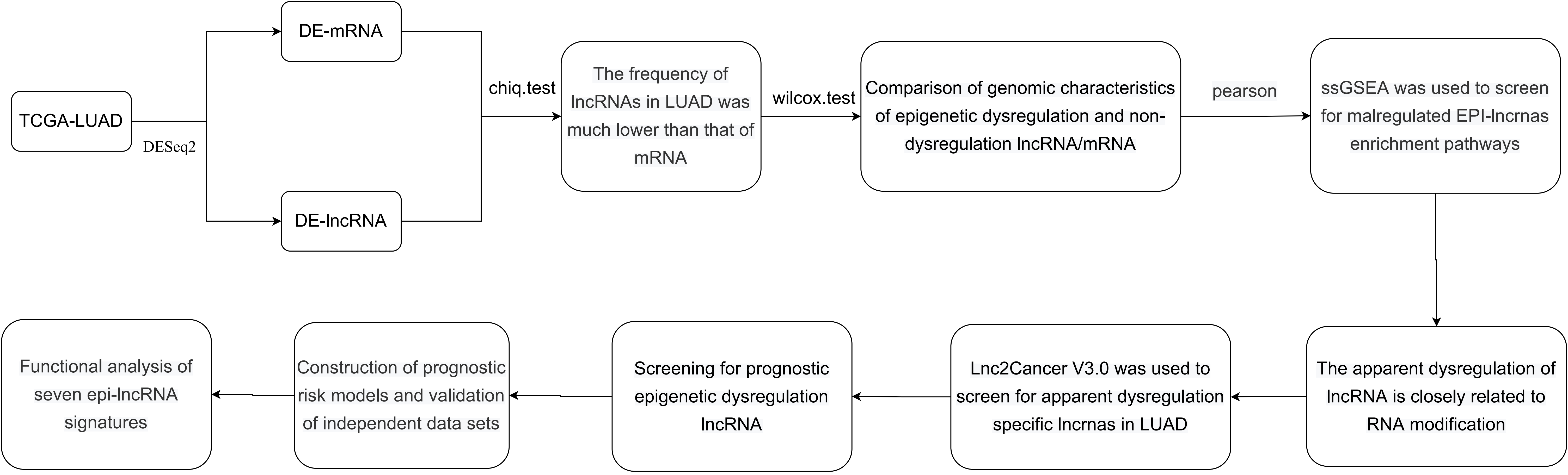
TCGA-LUAD
DESeq2
DE-mRNA
DE-lncRNA
chiq.test
The frequency of lncRNAs in LUAD was much lower than that of mRNA
wilcox.test
Comparison of genomic characteristics of epigenetic dysregulation and non-dysregulation lncRNA/mRNA
pearson
ssGSEA was used to screen for malregulated EPI-lncrnas enrichment pathways
The apparent dysregulation of lncRNA is closely related to RNA modification
Lnc2Cancer V3.0 was used to screen for apparent dysregulation specific lncrnas in LUAD
Screening for prognostic epigenetic dysregulation lncRNA
Construction of prognostic risk models and validation of independent data sets
Functional analysis of seven epi-lncRNA signatures

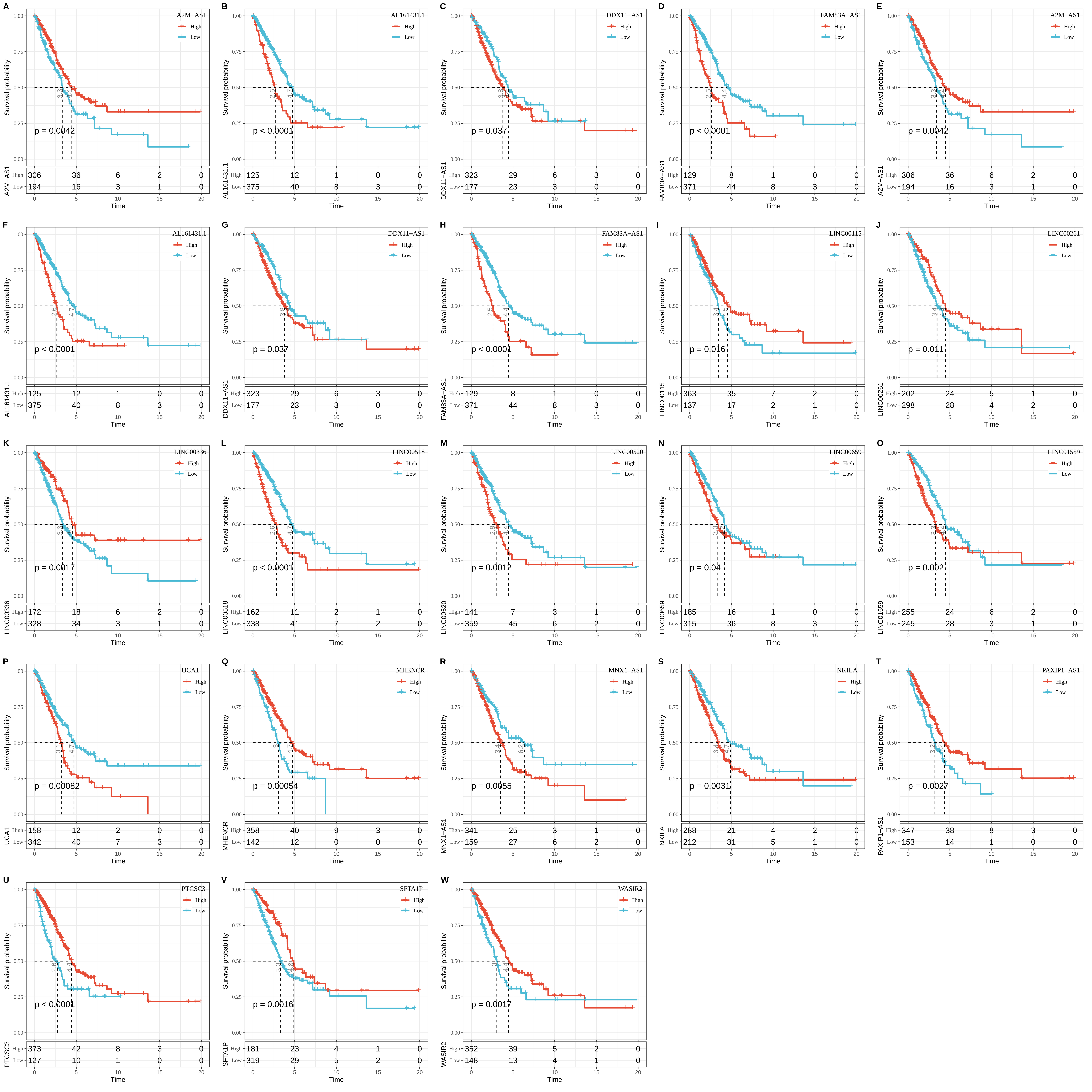

A A2M−AS1 p = 0.0042
B AL161431.1 p < 0.0001
C DDX11−AS1 p = 0.037
D FAM83A−AS1 p < 0.0001
E A2M−AS1 p = 0.0042
F AL161431.1 p < 0.0001
G DDX11−AS1 p = 0.037
H FAM83A−AS1 p < 0.0001
I LINC00115 p = 0.016
J LINC00261 p = 0.011
K LINC00336 p = 0.0017
L LINC00518 p < 0.0001
M LINC00520 p = 0.0012
N LINC00659 p = 0.04
O LINC01559 p = 0.002
P UCA1 p = 0.00082
Q MHENCR p = 0.00054
R MNX1−AS1 p = 0.0055
S NKILA p = 0.0031
T PAXIP1−AS1 p = 0.0027
U PTCSC3 p < 0.0001
V SFTA1P p = 0.0016
W WASIR2 p = 0.0017
High
Low
Survival probability
Time

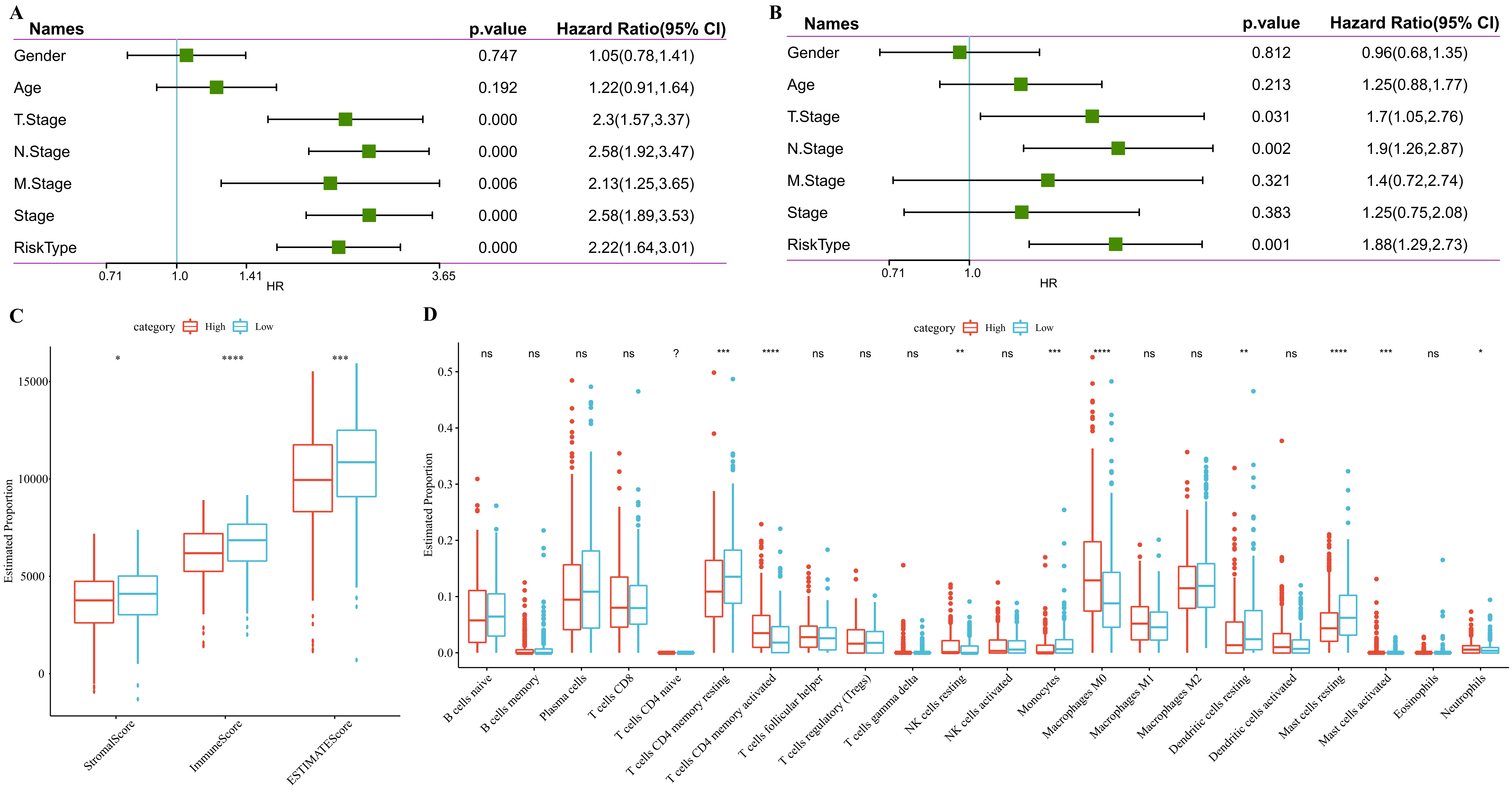
A
Names
p.value
Hazard Ratio(95% CI)
Gender 0.747 1.05(0.78,1.41)
Age 0.192 1.22(0.91,1.64)
T.Stage 0.000 2.3(1.57,3.37)
N.Stage 0.000 2.58(1.92,3.47)
M.Stage 0.006 2.13(1.25,3.65)
Stage 0.000 2.58(1.89,3.53)
RiskType 0.000 2.22(1.64,3.01)
0.71
1.0
1.41
3.65
HR
B
Names
p.value
Hazard Ratio(95% CI)
Gender 0.812 0.96(0.68,1.35)
Age 0.213 1.25(0.88,1.77)
T.Stage 0.031 1.7(1.05,2.76)
N.Stage 0.002 1.9(1.26,2.87)
M.Stage 0.321 1.4(0.72,2.74)
Stage 0.383 1.25(0.75,2.08)
RiskType 0.001 1.88(1.29,2.73)
0.71
1.0
HR
C
category High Low
Estimated Proportion
StromalScore
ImmuneScore
ESTIMATEScore
D
category High Low
Estimated Proportion
B cells naive
B cells memory
Plasma cells
T cells CD8
T cells CD4 naive
T cells CD4 memory resting
T cells CD4 memory activated
T cells follicular helper
T cells regulatory (Tregs)
T cells gamma delta
NK cells resting
NK cells activated
Monocytes
Macrophages M0
Macrophages M1
Macrophages M2
Dendritic cells resting
Dendritic cells activated
Mast cells resting
Mast cells activated
Eosinophils
Neutrophils

Supplementary Table1: Clinical characteristics of each sample in TCGA cohort

Samples	OS.time	OS	T.Stage	N.Stage	M.Stage	Stage	Gender	Age	RiskScore	type	Age
TCGA-05-4249-01	1523	0	T2	N0	M0	I	Male	67	-0.08594	Low	>65
TCGA-05-4250-01	121	1	T3	N1	M0	III	Female	79	1.833417	High	>65
TCGA-05-4382-01	607	0	T2	N0	M0	I	Male	68	-1.14446	Low	>65
TCGA-05-4384-01	426	0	T2	N2	M0	III	Male	66	0.108449	High	>65
TCGA-05-4389-01	1369	0	T1	N0	M0	I	Male	70	-0.16928	Low	>65
TCGA-05-4390-01	1126	0	T2	N0	M0	I	Female	58	0.628439	High	<=65
TCGA-05-4396-01	303	1	T4	N1	M0	III	Male	76	1.350493	High	>65
TCGA-05-4397-01	731	1	T2	N1	M0	II	Male	65	-0.19554	Low	<=65
TCGA-05-4398-01	1431	0	T4	N3	M0	III	Female	47	0.551347	High	<=65
TCGA-05-4402-01	244	1	T2	NA	M1	IV	Female	57	0.770154	High	<=65
TCGA-05-4403-01	578	0	T2	N0	M0	I	Male	76	0.544537	High	>65
TCGA-05-4405-01	610	0	T2	N0	M0	I	Female	74	0.209317	High	>65
TCGA-05-4415-01	91	1	T4	N2	M0	III	Male	57	1.309491	High	<=65

TCGA-05-4417-01	455	0	T2	N0	M0	I	Female	51	0.431007	High	<=65
TCGA-05-4418-01	274	1	T3	N2	M0	III	Male	69	2.007099	High	>65
TCGA-05-4420-01	912	0	T2	N0	M0	I	Male	41	-0.7381	Low	<=65
TCGA-05-4422-01	365	0	T2	N0	M0	I	Male	68	-1.90465	Low	>65
TCGA-05-4424-01	913	0	T3	N0	M0	II	Male	70	0.355888	High	>65
TCGA-05-4425-01	669	0	T2	N0	M1	IV	Female	70	0.336726	High	>65
TCGA-05-4426-01	791	0	T2	N0	M0	I	Male	71	-0.09056	Low	>65
TCGA-05-4427-01	791	0	T2	N1	M0	II	Female	65	0.536338	High	<=65
TCGA-05-4430-01	761	0	T2	N0	M0	I	Female	59	0.060791	High	<=65
TCGA-05-4432-01	761	0	T2	N1	M0	II	Male	66	-0.26065	Low	>65
TCGA-05-4433-01	730	0	T2	N0	M0	I	Male	82	-0.51265	Low	>65
TCGA-05-4434-01	457	1	T4	N1	M1	IV	Female	67	1.544767	High	>65
TCGA-05-5420-01	457	0	T2	N2	M0	III	Male	67	-0.67141	Low	>65
TCGA-05-5423-01	151	0	T2	N1	M0	II	Male	65	-1.0176	Low	<=65

TCGA-05-5425-01	882	0	T2	N1	M0	II	Male	68	0.571536	High	>65
TCGA-05-5428-01	670	0	T1	N1	M0	II	Male	57	-0.17285	Low	<=65
TCGA-05-5429-01	275	1	T3	N2	M0	III	Male	60	0.689953	High	<=65
TCGA-05-5715-01	62	0	T2	N0	M0	I	Female	69	0.357315	High	>65
TCGA-35-3615-01	14	0	T2	N0	M0	I	Male	57	0.089663	High	<=65
TCGA-35-4122-01	225	0	T1	N0	M0	I	Male	69	0.456503	High	>65
TCGA-35-4123-01	182	0	T1	N0	M0	I	Male	38	0.376225	High	<=65
TCGA-35-5375-01	264	0	T2	N2	M0	III	Male	61	0.051805	High	<=65
TCGA-38-4625-01	2973	0	T2	N0	M0	I	Female	66	-0.04034	Low	>65
TCGA-38-4626-01	3674	0	T2	N0	M0	NA	Female	57	-1.6052	Low	<=65
TCGA-38-4627-01	1147	1	T1	N1	M0	II	Female	64	-0.45792	Low	<=65
TCGA-38-4628-01	1492	1	T2	N1	M0	II	Female	65	1.236454	High	<=65
TCGA-38-4629-01	864	1	T3	N0	M0	II	Male	68	2.51656	High	>65
TCGA-38-4630-01	1073	1	T2	N0	M0	I	Female	75	-1.1423	Low	>65

TCGA-38-4631-01	354	1	T2	N0	M0	I	Female	72	-0.04565	Low	>65
TCGA-38-4632-01	1357	1	T2	N1	M1	IV	Male	42	0.523109	High	<=65
TCGA-38-6178-01	448	0	T2	N2	NA	III	Female	70	-0.01723	Low	>65
TCGA-38-7271-01	800	1	T1	N0	M0	I	Female	72	-0.74106	Low	>65
TCGA-38-A44F-01	133	0	T2	N0	M0	I	Male	80	-0.77416	Low	>65
TCGA-44-2655-01	1324	0	T1	N0	M0	I	Female	65	0.068493	High	<=65
TCGA-44-2656-01	1429	0	T2	N0	M0	I	Male	59	-0.52523	Low	<=65
TCGA-44-2657-01	1351	0	T2	NA	M0	I	Female	74	-0.98018	Low	>65
TCGA-44-2659-01	1367	0	T1	N1	M0	II	Female	65	-0.08377	Low	<=65
TCGA-44-2661-01	1159	0	T1	N0	M0	I	Female	69	-1.30512	Low	>65
TCGA-44-2662-01	1280	0	T2	N0	M0	I	Male	65	-0.46618	Low	<=65
TCGA-44-2665-01	1301	0	T2	N1	M0	II	Female	55	-1.31434	Low	<=65
TCGA-44-2666-01	97	1	T2	N0	M0	I	Male	43	-0.13424	Low	<=65
TCGA-44-2668-01	761	1	T2	N0	M0	I	Male	51	0.152386	High	<=65

TCGA-44-3396-01	1130	0	T2	N2	M0	III	Female	74	0.256723	High	>65
TCGA-44-3398-01	1163	0	T1	N0	M0	I	Female	77	-1.14637	Low	>65
TCGA-44-3917-01	1183	0	T2	N0	M0	I	Female	33	-0.67528	Low	<=65
TCGA-44-3918-01	1036	0	T1	N0	M0	I	Female	60	0.268087	High	<=65
TCGA-44-3919-01	1026	1	T1	N0	M0	I	Female	71	0.560864	High	>65
TCGA-44-4112-01	808	1	T2	N0	M0	I	Female	60	-0.28488	Low	<=65
TCGA-44-5643-01	1013	0	T2	N2	M0	III	Male	53	0.00027	High	<=65
TCGA-44-5644-01	863	0	T2	N0	NA	I	Female	51	0.872352	High	<=65
TCGA-44-5645-01	852	0	T1	NA	NA	I	Female	61	-2.26614	Low	<=65
TCGA-44-6145-01	595	0	T1	N0	M0	I	Female	62	0.531419	High	<=65
TCGA-44-6146-01	728	0	T3	N0	M0	II	Male	64	-1.07328	Low	<=65
TCGA-44-6147-01	845	0	T1	NA	M0	I	Female	67	-1.08511	Low	>65
TCGA-44-6148-01	704	0	T1	N0	M0	I	Male	60	-1.93324	Low	<=65
TCGA-44-6774-01	658	0	T1	N2	M0	III	Female	56	-0.47515	Low	<=65

TCGA-44-6775-01	705	0	T2	N0	NA	I	Female	72	-1.34064	Low	>65
TCGA-44-6776-01	2616	0	T1	N0	NA	I	Female	60	0.092862	High	<=65
TCGA-44-6777-01	987	1	T2	NA	NA	I	Female	85	0.26951	High	>65
TCGA-44-6778-01	1864	0	T1	N0	NA	I	Male	59	-1.28796	Low	<=65
TCGA-44-6779-01	500	1	T2	N1	NA	II	Female	50	2.434535	High	<=65
TCGA-44-7659-01	691	0	T1	N0	NA	I	Male	70	-0.11902	Low	>65
TCGA-44-7660-01	592	0	T2	N0	NA	I	Male	72	0.805952	High	>65
TCGA-44-7661-01	557	1	T2	N0	M0	I	Female	69	1.981673	High	>65
TCGA-44-7662-01	218	0	T2	N0	NA	I	Male	61	0.432503	High	<=65
TCGA-44-7667-01	1097	0	T3	N0	NA	II	Female	49	-0.38088	Low	<=65
TCGA-44-7669-01	574	1	T1	N1	NA	II	Male	59	0.166244	High	<=65
TCGA-44-7670-01	882	0	T1	N1	M0	II	Female	47	0.991491	High	<=65
TCGA-44-7671-01	889	0	T2	N0	M0	I	Male	64	0.042874	High	<=65
TCGA-44-7672-01	719	0	T1	N0	M0	I	Female	52	0.531457	High	<=65

TCGA-44-8117-01	385	0	T2	N0	M0	I	Female	54	1.320858	High	<=65
TCGA-44-8119-01	285	0	T3	N0	M0	II	Male	73	0.217839	High	>65
TCGA-44-8120-01	260	0	T2	N0	M0	I	Male	58	0.877334	High	<=65
TCGA-44-A479-01	486	0	T2	N0	NA	I	Female	73	-1.24157	Low	>65
TCGA-44-A47A-01	466	0	T2	N0	NA	I	Female	78	-0.57124	Low	>65
TCGA-44-A47B-01	287	0	T2	N0	M0	I	Male	79	-0.6101	Low	>65
TCGA-44-A47G-01	351	0	T1	N0	M0	I	Female	73	-1.01048	Low	>65
TCGA-44-A4SS-01	415	0	T1	N0	M0	I	Male	73	-0.26041	Low	>65
TCGA-44-A4SU-01	409	1	T1	N0	NA	I	Female	67	-1.04923	Low	>65
TCGA-49-4486-01	2318	1	T1	N0	M0	I	Male	72	-1.25689	Low	>65
TCGA-49-4487-01	855	1	T1	N0	M0	I	Female	72	0.137155	High	>65
TCGA-49-4488-01	869	1	T1	N0	NA	I	Female	74	1.028633	High	>65
TCGA-49-4490-01	385	1	T3	N2	M0	III	Female	45	-0.4904	Low	<=65
TCGA-49-4494-01	1081	1	T3	N2	M0	III	Male	77	1.547383	High	>65

TCGA-49-4501-01	1421	1	T2	N0	M0	I	Female	67	-0.52284	Low	>65
TCGA-49-4505-01	428	1	T2	N1	M0	II	Female	61	0.521741	High	<=65
TCGA-49-4506-01	999	1	T2	N1	M0	II	Female	68	1.059205	High	>65
TCGA-49-4507-01	268	1	T3	N1	M0	III	Female	73	-0.34533	Low	>65
TCGA-49-4510-01	896	1	T2	N1	M0	II	Female	51	-0.21286	Low	<=65
TCGA-49-4512-01	905	1	T2	N2	NA	III	Female	69	-0.1434	Low	>65
TCGA-49-4514-01	1700	0	T1	N0	M0	I	Female	79	0.007032	High	>65
TCGA-49-6742-01	488	1	T2	N1	M0	II	Male	70	0.924856	High	>65
TCGA-49-6743-01	1621	0	T1	N2	NA	III	Female	81	0.038269	High	>65
TCGA-49-6744-01	1683	0	T2	N1	NA	II	Female	64	0.029266	High	<=65
TCGA-49-6745-01	522	0	T2	N2	M0	III	Male	82	1.452148	High	>65
TCGA-49-6761-01	354	0	T1	N2	NA	III	Female	68	0.677134	High	>65
TCGA-49-6767-01	677	0	T3	N0	NA	II	Female	46	1.529972	High	<=65
TCGA-49-AAQV-01	677	1	T1	N1	NA	II	Female	63	-0.07626	Low	<=65

TCGA-49-AAR0-01	4765	0	T1	N0	NA	I	Male	57	-1.14397	Low	<=65
TCGA-49-AAR2-01	2224	0	T2	N0	NA	I	Male	64	-2.3627	Low	<=65
TCGA-49-AAR3-01	1893	0	T2	N1	NA	II	Male	69	1.685501	High	>65
TCGA-49-AAR4-01	879	1	T2	N2	NA	III	Male	51	-0.33311	Low	<=65
TCGA-49-AAR9-01	260	1	T3	N0	NA	II	Male	61	2.604757	High	<=65
TCGA-49-AARE-01	1229	1	T1	N0	NA	I	Female	51	-0.33759	Low	<=65
TCGA-49-AARN-01	1135	1	T1	N0	NA	I	Female	56	0.010654	High	<=65
TCGA-49-AARO-01	3759	0	T1	N0	NA	I	Female	39	-0.05456	Low	<=65
TCGA-49-AARQ-01	6732	0	T2	N0	NA	I	Female	41	-0.64417	Low	<=65
TCGA-49-AARR-01	4992	0	T1	N0	NA	I	Male	68	-1.81395	Low	>65
TCGA-4B-A93V-01	300	1	T1	N0	M0	I	Female	52	0.038066	High	<=65
TCGA-50-5044-01	624	1	T4	N1	M0	III	Female	72	1.743792	High	>65
TCGA-50-5045-01	2174	1	T2	N1	M0	NA	Female	57	-0.29596	Low	<=65
TCGA-50-5049-01	3094	0	T2	N0	M0	I	Male	70	-1.02242	Low	>65

TCGA-50-5051-01	478	1	T2	N2	M0	III	Female	42	0.217378	High	<=65
TCGA-50-5055-01	1830	1	T1	N1	M0	II	Female	79	-1.04831	Low	>65
TCGA-50-5066-01	1442	0	T2	N0	M0	I	Male	72	-0.08018	Low	>65
TCGA-50-5068-01	1499	1	T2	N1	NA	II	Female	59	-0.53101	Low	<=65
TCGA-50-5072-01	250	1	T2	N2	M0	III	Male	74	2.333882	High	>65
TCGA-50-5930-01	282	1	T2	N2	M0	III	Male	47	1.082704	High	<=65
TCGA-50-5931-01	434	1	T2	N0	M0	I	Female	75	-1.78505	Low	>65
TCGA-50-5932-01	1235	1	T2	N1	M0	II	Male	75	0.216083	High	>65
TCGA-50-5933-01	2393	1	T4	N2	M0	III	Male	72	2.121544	High	>65
TCGA-50-5935-01	653	1	T1	N0	M0	I	Female	86	0.146578	High	>65
TCGA-50-5936-01	257	1	T2	N2	M0	III	Male	58	1.317266	High	<=65
TCGA-50-5939-01	460	1	T2	N0	M0	I	Male	85	1.757679	High	>65
TCGA-50-5941-01	1474	0	T2	N2	M0	III	Female	55	0.270344	High	<=65
TCGA-50-5942-01	1847	0	T1	N0	M0	I	Female	67	-0.92887	Low	>65

TCGA-50-5944-01	1750	0	T1	N0	M0	I	Female	69	-1.29022	Low	>65
TCGA-50-5946-01	1617	0	T1	N0	NA	I	Male	62	0.728351	High	<=65
TCGA-50-6590-01	1288	1	T2	N0	M0	I	Female	72	1.365511	High	>65
TCGA-50-6591-01	119	1	T2	N0	M1	IV	Female	63	0.404321	High	<=65
TCGA-50-6592-01	777	1	T2	N0	M0	I	Female	71	0.86926	High	>65
TCGA-50-6593-01	336	1	T1	N2	M0	III	Female	49	0.265193	High	<=65
TCGA-50-6594-01	370	1	T3	N2	M0	III	Female	79	1.995196	High	>65
TCGA-50-6595-01	189	1	T2	N2	M0	III	Female	74	1.770598	High	>65
TCGA-50-6597-01	1268	1	T2	N0	M0	I	Female	79	0.804853	High	>65
TCGA-50-6673-01	22	1	T1	N0	M0	I	Female	84	1.017087	High	>65
TCGA-50-7109-01	308	1	T1	N0	M0	I	Male	60	-0.46523	Low	<=65
TCGA-50-8457-01	1125	0	T1	N0	M0	I	Female	63	-0.97642	Low	<=65
TCGA-50-8459-01	1119	0	T3	N0	M0	II	Male	68	-0.87747	Low	>65
TCGA-50-8460-01	829	0	T1	N0	M0	I	Male	74	-0.73553	Low	>65

TCGA-53-7624-01	1043	1	T2	N0	M1	IV	Female	40	2.15905	High	<=65
TCGA-53-7626-01	929	1	T1	N1	M0	II	Female	76	-1.24277	Low	>65
TCGA-53-7813-01	424	0	T4	N0	M0	III	Female	51	0.133455	High	<=65
TCGA-53-A4EZ-01	1071	0	T2	N1	NA	II	Male	63	-0.36616	Low	<=65
TCGA-55-1592-01	701	1	T2	N0	M0	I	Male	65	-1.63535	Low	<=65
TCGA-55-1594-01	1178	0	T2	N2	M0	III	Male	68	-1.34152	Low	>65
TCGA-55-1596-01	2065	0	T2	N1	M0	II	Male	55	0.168366	High	<=65
TCGA-55-5899-01	930	0	T1	N1	M0	NA	Male	58	-0.0713	Low	<=65
TCGA-55-6543-01	435	0	T1	N0	NA	I	Female	60	0.00048	High	<=65
TCGA-55-6642-01	2449	0	T2	N0	NA	I	Male	63	0.031014	High	<=65
TCGA-55-6712-01	171	1	T2	N1	NA	II	Male	71	1.723774	High	>65
TCGA-55-6968-01	1293	1	T1	N0	M1	IV	Male	61	0.101656	High	<=65
TCGA-55-6970-01	464	1	T2	N2	NA	III	Female	67	0.946433	High	>65
TCGA-55-6971-01	1400	0	T2	N0	NA	I	Female	59	0.06032	High	<=65

TCGA-55-6972-01	1632	1	T2	N0	M0	I	Male	72	-1.66421	Low	>65
TCGA-55-6975-01	118	1	T2	N1	M0	II	Male	61	3.459865	High	<=65
TCGA-55-6978-01	176	1	T2	N0	NA	II	Male	81	0.367624	High	>65
TCGA-55-6979-01	237	1	T2	N1	M0	II	Female	59	0.254283	High	<=65
TCGA-55-6980-01	2109	0	T1	N0	M0	I	Male	56	0.139221	High	<=65
TCGA-55-6981-01	1379	1	T1	N2	M0	III	Female	53	1.145543	High	<=65
TCGA-55-6982-01	995	1	T2	N1	M0	II	Female	79	1.597566	High	>65
TCGA-55-6983-01	2823	0	T2	N1	M0	II	Male	81	0.763027	High	>65
TCGA-55-6984-01	760	1	T2	N1	M0	II	Female	71	0.607015	High	>65
TCGA-55-6985-01	1233	0	T2	N0	NA	I	Female	58	0.556033	High	<=65
TCGA-55-6986-01	3261	0	T2	N0	M0	I	Female	74	-0.37987	Low	>65
TCGA-55-6987-01	2137	0	T1	N0	M0	I	Male	77	0.459362	High	>65
TCGA-55-7227-01	952	1	T3	N1	NA	III	Male	77	-0.21488	Low	>65
TCGA-55-7281-01	872	0	T1	N0	M0	I	Female	70	0.550378	High	>65

TCGA-55-7283-01	609	0	T3	N2	NA	III	Female	76	0.407272	High	>65
TCGA-55-7284-01	243	1	T3	N0	NA	II	Male	74	-0.24462	Low	>65
TCGA-55-7570-01	824	0	T1	N0	NA	I	Male	60	-0.28393	Low	<=65
TCGA-55-7573-01	487	0	T1	N0	NA	I	Female	72	-1.25562	Low	>65
TCGA-55-7574-01	995	1	T2	N0	M0	I	Female	64	-0.63559	Low	<=65
TCGA-55-7576-01	670	0	T2	N0	M0	I	Male	54	1.180865	High	<=65
TCGA-55-7724-01	705	0	T2	N0	NA	I	Female	76	-0.04707	Low	>65
TCGA-55-7725-01	442	0	T1	N0	NA	I	Female	68	-0.81315	Low	>65
TCGA-55-7726-01	652	0	T1	N0	NA	I	Female	72	1.495081	High	>65
TCGA-55-7727-01	119	0	T1	N2	NA	III	Male	70	0.568256	High	>65
TCGA-55-7728-01	704	0	T2	N0	NA	I	Female	64	-1.1685	Low	<=65
TCGA-55-7815-01	773	0	T2	N0	NA	I	Male	76	-0.17608	Low	>65
TCGA-55-7816-01	468	1	TX	NA	NA	IV	Female	49	-1.6315	Low	<=65
TCGA-55-7903-01	567	0	T1	N0	NA	I	Male	64	0.510783	High	<=65

TCGA-55-7907-01	343	1	T2	N1	NA	II	Male	77	1.193886	High	>65
TCGA-55-7910-01	1040	0	T2	N0	M0	II	Female	50	0.277149	High	<=65
TCGA-55-7911-01	537	0	T1	N0	NA	I	Female	70	0.536726	High	>65
TCGA-55-7913-01	561	1	T1	N0	NA	I	Female	61	1.302649	High	<=65
TCGA-55-7914-01	187	1	T1	N1	NA	II	Female	71	-0.24179	Low	>65
TCGA-55-7994-01	603	0	T3	N0	NA	II	Male	81	0.185486	High	>65
TCGA-55-7995-01	889	0	T1	N0	M0	I	Female	73	-0.92365	Low	>65
TCGA-55-8085-01	904	0	T1	N0	M0	I	Male	64	0.037387	High	<=65
TCGA-55-8087-01	462	0	T2	N0	NA	I	Female	59	-1.52415	Low	<=65
TCGA-55-8089-01	702	1	T1	N0	M0	I	Male	56	1.608353	High	<=65
TCGA-55-8090-01	598	1	T1	N0	M0	I	Male	80	0.798316	High	>65
TCGA-55-8091-01	600	0	T2	N0	NA	I	Male	74	-0.49117	Low	>65
TCGA-55-8092-01	154	1	T3	N0	NA	II	Male	75	0.12232	High	>65
TCGA-55-8094-01	541	0	T2	N0	M1	IV	Male	51	0.698311	High	<=65

TCGA-55-8096-01	719	1	T2	N0	NA	I	Female	67	-0.53194	Low	>65
TCGA-55-8097-01	476	0	T1	N0	NA	I	Female	60	-1.25938	Low	<=65
TCGA-55-8203-01	547	0	T1	N0	M0	I	Female	69	0.86674	High	>65
TCGA-55-8204-01	515	0	T2	N0	NA	I	Female	87	0.657937	High	>65
TCGA-55-8205-01	599	0	T2	N0	M0	II	Female	76	-0.03896	Low	>65
TCGA-55-8206-01	888	0	T1	N0	M0	I	Male	56	-1.90874	Low	<=65
TCGA-55-8207-01	977	0	T2	N0	NA	I	Male	73	-0.77193	Low	>65
TCGA-55-8208-01	674	0	T1	N0	M0	I	Female	73	0.280021	High	>65
TCGA-55-8299-01	469	1	T1	N0	NA	I	Female	61	0.890824	High	<=65
TCGA-55-8301-01	534	0	T2	N0	NA	I	Male	58	-0.42403	Low	<=65
TCGA-55-8302-01	478	0	T2	N0	NA	I	Male	54	0.888736	High	<=65
TCGA-55-8505-01	440	0	T1	N2	NA	III	Male	62	1.063756	High	<=65
TCGA-55-8506-01	11	0	T3	N0	NA	II	Female	62	0.933457	High	<=65
TCGA-55-8507-01	418	0	T1	N0	NA	I	Male	53	0.185273	High	<=65

TCGA-55-8508-01	617	0	T2	N1	NA	II	Female	60	0.026936	High	<=65
TCGA-55-8510-01	539	0	T2	N0	NA	I	Female	55	-0.08152	Low	<=65
TCGA-55-8511-01	552	0	T2	N0	NA	I	Female	73	1.05352	High	>65
TCGA-55-8512-01	607	1	T1	N1	M1	IV	Male	41	-1.36234	Low	<=65
TCGA-55-8513-01	791	0	T3	N0	NA	II	Female	77	-1.65993	Low	>65
TCGA-55-8514-01	520	0	T2	N0	NA	I	Female	70	-0.59903	Low	>65
TCGA-55-8614-01	536	0	T2	N0	NA	I	Male	76	0.299271	High	>65
TCGA-55-8615-01	446	0	T3	N2	NA	III	Male	67	-0.01252	Low	>65
TCGA-55-8616-01	48	0	T2	N0	M0	I	Female	58	0.022544	High	<=65
TCGA-55-8619-01	416	0	T3	N0	NA	II	Female	72	-1.70339	Low	>65
TCGA-55-8620-01	375	1	T1	N1	M1	IV	Male	60	-1.14875	Low	<=65
TCGA-55-8621-01	515	0	T1	N0	NA	I	Female	75	-0.5269	Low	>65
TCGA-55-A48X-01	689	0	T1	N1	M0	II	Female	63	-0.13245	Low	<=65
TCGA-55-A48Y-01	630	0	T2	N0	M0	II	Male	69	1.314141	High	>65

TCGA-55-A48Z-01	651	0	T1	N3	NA	III	Female	60	-0.41033	Low	<=65
TCGA-55-A490-01	99	1	T2	N0	NA	II	Male	78	0.853268	High	>65
TCGA-55-A491-01	626	0	T1	N0	NA	I	Female	81	0.925117	High	>65
TCGA-55-A492-01	596	0	T1	N0	NA	I	Female	70	-1.73168	Low	>65
TCGA-55-A493-01	28	0	T2	N0	M0	I	Female	54	0.242372	High	<=65
TCGA-55-A494-01	481	0	T2	N0	NA	I	Female	61	1.466487	High	<=65
TCGA-55-A4DF-01	440	1	T1	N0	NA	I	Male	88	-0.44793	Low	>65
TCGA-55-A4DG-01	608	0	T1	N0	NA	I	Male	71	-0.65827	Low	>65
TCGA-55-A57B-01	546	0	T1	N0	M0	I	Female	80	-0.86884	Low	>65
TCGA-62-8394-01	139	1	T4	N2	M0	III	Female	65	0.81324	High	<=65
TCGA-62-8395-01	1216	0	T3	N0	M0	II	Female	80	-0.38317	Low	>65
TCGA-62-8397-01	1289	0	T3	N0	M0	II	Female	70	-1.42539	Low	>65
TCGA-62-8398-01	444	1	T2	N2	M0	III	Male	55	1.699525	High	<=65
TCGA-62-8399-01	2696	0	T2	N2	M0	III	Male	62	-0.9361	Low	<=65

TCGA-62-8402-01	1498	1	T2	N2	M0	III	Female	73	0.123667	High	>65
TCGA-62-A46O-01	1454	1	T2	N0	M0	I	Female	65	1.461176	High	<=65
TCGA-62-A46P-01	594	1	T2	N0	M0	I	Male	65	0.228957	High	<=65
TCGA-62-A46R-01	1725	1	T2	N0	M0	I	Female	54	-0.7228	Low	<=65
TCGA-62-A46S-01	1653	1	T2	N0	M0	I	Male	73	-0.59911	Low	>65
TCGA-62-A46V-01	2199	0	T2	N0	M0	I	Female	78	-0.16031	Low	>65
TCGA-62-A46Y-01	414	1	T2	N2	M0	III	Female	70	-0.55372	Low	>65
TCGA-62-A470-01	1194	1	T2	N0	M0	I	Male	84	-1.16831	Low	>65
TCGA-62-A471-01	1246	0	T2	N1	M0	II	Male	64	2.833768	High	<=65
TCGA-62-A472-01	910	0	T3	N0	M0	II	Male	70	0.223337	High	>65
TCGA-64-1676-01	1728	0	T1	N0	M0	I	Male	58	-0.4431	Low	<=65
TCGA-64-1677-01	628	1	T2	N2	M0	III	Female	77	0.037608	High	>65
TCGA-64-1678-01	1189	0	T2	N0	M0	NA	Female	70	-0.33285	Low	>65
TCGA-64-1679-01	2488	0	T1	N2	M0	III	Female	58	0.609693	High	<=65

TCGA-64-1680-01	1126	0	T2	N2	M1	IV	Male	63	0.375606	High	<=65
TCGA-64-1681-01	1167	1	T1	N0	M0	I	Female	61	-0.47728	Low	<=65
TCGA-64-5774-01	2676	0	T2	N0	M0	I	Male	60	0.112763	High	<=65
TCGA-64-5775-01	62	1	T4	N0	M0	III	Male	71	1.834004	High	>65
TCGA-64-5778-01	1305	0	T2	N0	M0	I	Male	60	-0.58058	Low	<=65
TCGA-64-5779-01	864	0	T2	N2	M0	III	Male	61	-0.83089	Low	<=65
TCGA-64-5781-01	1559	0	T2	N0	M0	I	Female	55	1.104262	High	<=65
TCGA-64-5815-01	866	0	T2	N1	M0	II	Male	74	0.731592	High	>65
TCGA-67-3770-01	610	0	T1	N0	M0	I	Female	70	-1.23092	Low	>65
TCGA-67-3771-01	610	0	T1	N0	M0	I	Female	77	-1.01738	Low	>65
TCGA-67-3772-01	573	0	T2	N0	M0	I	Female	82	-0.61581	Low	>65
TCGA-67-3773-01	427	0	T2	N0	M0	I	Female	84	-0.91136	Low	>65
TCGA-67-3774-01	385	0	T2	N0	M0	I	Female	73	-0.48421	Low	>65
TCGA-67-4679-01	448	0	T3	N0	M0	NA	Male	69	-1.15183	Low	>65

TCGA-67-6215-01	174	0	T2	N0	M0	I	Female	52	-0.48455	Low	<=65
TCGA-67-6216-01	141	0	T1	N0	M0	I	Female	57	-0.78028	Low	<=65
TCGA-67-6217-01	422	0	T2	N1	M0	II	Female	73	-0.1256	Low	>65
TCGA-69-7760-01	202	0	T3	N0	M0	II	Male	73	1.632091	High	>65
TCGA-69-7761-01	186	0	T2	N0	NA	I	Male	84	-0.32956	Low	>65
TCGA-69-7763-01	690	0	T1	N0	M0	I	Male	69	-0.31219	Low	>65
TCGA-69-7764-01	414	0	T1	N0	M0	I	Male	75	-1.01119	Low	>65
TCGA-69-7765-01	165	0	T4	N0	NA	NA	Male	56	-0.03797	Low	<=65
TCGA-69-7973-01	230	0	T2	N0	M0	I	Female	42	0.747246	High	<=65
TCGA-69-7974-01	184	0	T2	N2	NA	III	Female	54	1.286737	High	<=65
TCGA-69-7978-01	134	0	T2	N1	NA	II	Male	59	0.726066	High	<=65
TCGA-69-7979-01	408	0	T2	N0	NA	I	Female	71	-0.39067	Low	>65
TCGA-69-7980-01	411	0	T1	N0	M0	I	Female	70	-1.11649	Low	>65
TCGA-69-8253-01	426	0	T1	N1	NA	II	Female	59	0.094546	High	<=65

TCGA-69-8254-01	409	0	T2	NA	NA	NA	Male	85	0.167359	High	>65
TCGA-69-8255-01	129	0	T1	N0	M0	I	Male	71	-2.31772	Low	>65
TCGA-69-8453-01	813	0	T3	N0	NA	II	Male	77	-1.11111	Low	>65
TCGA-69-A59K-01	591	0	T3	N0	M0	II	Female	60	0.585701	High	<=65
TCGA-71-6725-01	256	0	T2	N0	M0	I	Female	48	0.819534	High	<=65
TCGA-71-8520-01	210	1	T2	N0	M0	I	Female	60	0.100625	High	<=65
TCGA-73-4658-01	1600	1	T2	N0	M0	I	Female	80	0.383038	High	>65
TCGA-73-4659-01	711	1	T2	N2	M0	III	Male	66	-0.24565	Low	>65
TCGA-73-4662-01	2515	0	T1	N0	M0	I	Female	65	-0.81589	Low	<=65
TCGA-73-4666-01	800	0	T1	N0	M1	IV	Female	52	1.586136	High	<=65
TCGA-73-4668-01	467	0	T2	N1	M0	II	Female	66	0.249916	High	>65
TCGA-73-4670-01	131	0	T2	N0	M1	IV	Female	69	1.83751	High	>65
TCGA-73-4675-01	922	1	T3	N1	M0	III	Male	59	-0.35825	Low	<=65
TCGA-73-4676-01	281	1	T2	N1	M0	II	Male	45	1.600832	High	<=65

TCGA-73-4677-01	38	1	T2	N0	M0	NA	Male	74	0.038448	High	>65
TCGA-73-7498-01	1189	0	T1	N0	M0	I	Female	58	-1.04978	Low	<=65
TCGA-73-7499-01	1531	1	T2	N0	M0	I	Female	81	0.568017	High	>65
TCGA-73-A9RS-01	340	1	T3	N0	M0	II	Male	41	0.997126	High	<=65
TCGA-75-5125-01	2027	1	T2	N1	M0	II	Male	NA	-0.20176	Low	NA
TCGA-75-5146-01	2368	0	T2	N0	M0	I	Male	NA	-1.26333	Low	NA
TCGA-75-5147-01	1333	0	T2	N0	M0	I	Female	NA	-0.07078	Low	NA
TCGA-75-6206-01	2590	0	T2	N0	M0	I	Male	NA	0.200957	High	NA
TCGA-75-6212-01	1516	1	T2	N1	M0	II	Female	NA	-1.05198	Low	NA
TCGA-75-6214-01	1115	1	T2	N2	M0	III	Female	NA	1.863079	High	NA
TCGA-75-7025-01	3305	0	T2	N0	M0	I	Male	NA	-1.65976	Low	NA
TCGA-75-7027-01	3059	0	T2	N0	M0	I	Male	NA	-0.05926	Low	NA
TCGA-78-7143-01	4961	1	T2	N0	M0	I	Female	62	0.492006	High	<=65
TCGA-78-7145-01	826	1	T4	N1	M1	IV	Female	52	1.300587	High	<=65

TCGA-78-7146-01	173	1	T2	N2	M0	III	Female	71	2.99426	High	>65
TCGA-78-7147-01	586	1	T2	N1	M0	II	Female	67	0.903247	High	>65
TCGA-78-7148-01	626	1	T2	N1	M0	II	Male	71	1.621021	High	>65
TCGA-78-7149-01	3940	0	T4	N0	M0	III	Male	71	-1.18238	Low	>65
TCGA-78-7150-01	666	1	T2	N1	M0	II	Male	59	2.085393	High	<=65
TCGA-78-7152-01	1215	1	T2	N0	M0	I	Male	65	0.134123	High	<=65
TCGA-78-7153-01	3635	0	T2	N0	M0	I	Female	65	-0.54463	Low	<=65
TCGA-78-7154-01	593	1	T3	N2	M0	III	Male	72	-1.44308	Low	>65
TCGA-78-7155-01	1171	1	T2	N0	M0	I	Male	68	-0.90968	Low	>65
TCGA-78-7156-01	976	1	T4	N1	M1	IV	Male	62	-0.85957	Low	<=65
TCGA-78-7158-01	179	1	T4	N2	M0	III	Female	59	0.192151	High	<=65
TCGA-78-7159-01	1974	0	T1	NA	M0	I	Female	60	0.588378	High	<=65
TCGA-78-7160-01	697	1	T4	N2	M1	IV	Male	61	-0.03201	Low	<=65
TCGA-78-7161-01	291	1	T3	N0	M0	II	Female	69	0.713055	High	>65

TCGA-78-7162-01	3169	1	T1	N0	M0	I	Male	75	-0.44706	Low	>65
TCGA-78-7163-01	7248	0	T2	N0	M0	I	Male	60	-0.47266	Low	<=65
TCGA-78-7166-01	258	1	T2	N1	M0	II	Male	84	1.71994	High	>65
TCGA-78-7167-01	2681	1	T2	N0	M1	IV	Male	77	-0.66926	Low	>65
TCGA-78-7220-01	807	1	T2	N2	M0	III	Female	53	1.993546	High	<=65
TCGA-78-7535-01	949	1	T2	N0	M0	I	Male	45	0.8198	High	<=65
TCGA-78-7536-01	244	1	T2	N2	M0	III	Male	69	1.332615	High	>65
TCGA-78-7537-01	1622	1	T2	N0	M0	I	Male	72	-0.84391	Low	>65
TCGA-78-7539-01	791	0	T2	N0	M0	II	Female	75	0.161138	High	>65
TCGA-78-7540-01	1197	1	T2	N0	M0	I	Female	66	0.205118	High	>65
TCGA-78-7542-01	321	1	T2	N0	M0	I	Male	56	1.571236	High	<=65
TCGA-78-7633-01	1528	1	T2	N0	M0	I	Male	67	0.433053	High	>65
TCGA-78-8640-01	7062	0	T1	N1	M0	II	Male	59	-1.15972	Low	<=65
TCGA-78-8648-01	1209	1	T3	N0	M0	II	Female	58	-1.03518	Low	<=65

TCGA-78-8655-01	2360	0	T1	N0	M0	I	Female	77	0.563668	High	>65
TCGA-78-8660-01	321	1	T2	N1	M0	II	Male	69	-0.5816	Low	>65
TCGA-78-8662-01	3361	1	T2	N0	M0	I	Female	53	-0.3732	Low	<=65
TCGA-80-5608-01	2832	0	T1	N0	M0	I	Female	NA	0.36946	High	NA
TCGA-80-5611-01	2595	0	T2	N0	M0	I	Male	NA	0.153298	High	NA
TCGA-83-5908-01	824	0	T1	N0	M0	I	Female	59	1.736105	High	<=65
TCGA-86-6562-01	376	1	T2	N1	M0	II	Male	52	0.712116	High	<=65
TCGA-86-6851-01	179	0	T1	N1	M0	II	Female	73	0.152285	High	>65
TCGA-86-7701-01	947	0	T2	N0	M1	IV	Male	66	1.08922	High	>65
TCGA-86-7711-01	1046	1	T2	N1	M0	II	Male	70	0.115383	High	>65
TCGA-86-7713-01	1157	0	T2	N0	M0	II	Male	70	0.443	High	>65
TCGA-86-7714-01	625	1	T1	N2	M0	III	Female	61	-0.62017	Low	<=65
TCGA-86-7953-01	997	0	T1	N0	M0	I	Female	69	0.37593	High	>65
TCGA-86-7954-01	605	0	T2	N0	M0	I	Female	68	-0.67034	Low	>65

TCGA-86-7955-01	1072	0	T2	N0	M0	I	Male	62	0.174407	High	<=65
TCGA-86-8054-01	1148	0	T2	N1	M0	II	Male	61	0.755889	High	<=65
TCGA-86-8055-01	124	1	T2	N1	M0	II	Male	79	-0.05124	Low	>65
TCGA-86-8056-01	139	0	T4	N0	M0	III	Female	63	-0.14486	Low	<=65
TCGA-86-8073-01	740	0	T2	N0	M0	I	Male	58	-0.91622	Low	<=65
TCGA-86-8074-01	24	0	T1	N1	M0	II	Female	62	0.188341	High	<=65
TCGA-86-8075-01	694	1	T2	N0	M0	I	Female	66	0.192116	High	>65
TCGA-86-8076-01	993	0	T1	N0	M0	I	Male	42	-0.16606	Low	<=65
TCGA-86-8278-01	944	0	T2	N1	M0	II	Female	63	0.487883	High	<=65
TCGA-86-8279-01	949	0	T2	N1	M0	II	Male	46	0.569492	High	<=65
TCGA-86-8280-01	701	0	T2	N0	M0	II	Female	54	-0.97304	Low	<=65
TCGA-86-8358-01	653	0	T2	N0	M0	I	Male	44	-0.0211	Low	<=65
TCGA-86-8359-01	444	1	T3	N2	M0	III	Male	52	0.358769	High	<=65
TCGA-86-8585-01	353	0	T2	N0	M0	I	Male	57	-0.61567	Low	<=65

TCGA-86-8668-01	423	0	T1	N0	M0	I	Female	61	-0.62507	Low	<=65
TCGA-86-8669-01	938	0	T1	N0	M0	I	Male	64	-1.17974	Low	<=65
TCGA-86-8671-01	839	0	T2	N1	M0	II	Female	72	-1.79856	Low	>65
TCGA-86-8672-01	19	1	T3	N0	M0	II	Male	59	0.699118	High	<=65
TCGA-86-8673-01	862	0	T2	N0	M0	I	Male	61	1.491277	High	<=65
TCGA-86-8674-01	806	0	T2	N1	M0	II	Male	50	0.381091	High	<=65
TCGA-86-A456-01	896	0	T1	N0	M0	I	Female	78	-0.29914	Low	>65
TCGA-86-A4D0-01	116	1	T2	N0	M0	II	Male	48	-0.61675	Low	<=65
TCGA-86-A4JF-01	737	1	T3	N0	M0	II	Male	56	0.307485	High	<=65
TCGA-86-A4P7-01	415	0	T2	N0	M0	I	Female	63	-1.1633	Low	<=65
TCGA-86-A4P8-01	805	0	T1	N2	NA	III	Female	59	-2.6647	Low	<=65
TCGA-91-6828-01	323	0	T1	N0	M0	I	Male	70	0.488976	High	>65
TCGA-91-6829-01	1258	1	T2	N0	NA	I	Male	78	0.786326	High	>65
TCGA-91-6830-01	60	0	T1	N1	NA	II	Female	65	1.297081	High	<=65

TCGA-91-6831-01	310	0	T2	N0	NA	I	Male	66	-0.73781	Low	>65
TCGA-91-6835-01	79	0	T1	N0	M0	I	Female	81	0.349322	High	>65
TCGA-91-6836-01	417	0	T2	N0	NA	I	Female	52	2.444818	High	<=65
TCGA-91-6840-01	372	0	T1	N0	M0	I	Female	59	-1.04947	Low	<=65
TCGA-91-6847-01	842	0	T2	N0	NA	I	Female	62	1.162224	High	<=65
TCGA-91-6848-01	224	0	T2	N2	NA	III	Male	59	0.543935	High	<=65
TCGA-91-6849-01	35	0	T2	N2	NA	III	Female	75	0.809188	High	>65
TCGA-91-7771-01	492	0	T3	N0	NA	II	Male	62	-1.20026	Low	<=65
TCGA-91-8496-01	505	0	T2	NA	NA	I	Female	63	-1.07383	Low	<=65
TCGA-91-8497-01	434	1	T1	N0	NA	I	Female	75	-1.5477	Low	>65
TCGA-91-8499-01	36	0	T1	N0	NA	I	Female	76	0.59405	High	>65
TCGA-91-A4BC-01	44	0	T2	N0	NA	II	Male	59	-1.32686	Low	<=65
TCGA-91-A4BD-01	603	0	T1	N1	NA	II	Male	78	-1.05881	Low	>65
TCGA-93-7347-01	683	0	T1	N0	NA	I	Female	76	-0.35084	Low	>65

TCGA-93-7348-01	531	0	T1	N0	NA	I	Female	75	-0.23199	Low	>65
TCGA-93-8067-01	186	0	T2	N0	NA	I	Male	77	0.953666	High	>65
TCGA-93-A4JN-01	718	0	T2	N0	M1	IV	Male	71	-0.83845	Low	>65
TCGA-93-A4JO-01	33	1	T1	N0	NA	I	Male	70	-0.74615	Low	>65
TCGA-93-A4JP-01	578	0	TX	NA	M1	IV	Male	64	-1.13605	Low	<=65
TCGA-93-A4JQ-01	526	0	T1	N0	NA	I	Male	49	-0.52	Low	<=65
TCGA-95-7039-01	1272	0	T3	N0	NA	II	Female	54	0.75684	High	<=65
TCGA-95-7043-01	503	1	T1	N0	NA	I	Female	63	0.437218	High	<=65
TCGA-95-7562-01	87	1	T2	N1	M0	II	Male	71	0.178929	High	>65
TCGA-95-7567-01	568	0	T2	N1	M0	II	Male	61	1.372623	High	<=65
TCGA-95-7944-01	377	0	T1	N0	M0	I	Male	71	0.44865	High	>65
TCGA-95-7947-01	477	0	T1	N0	M0	I	Male	67	-0.93494	Low	>65
TCGA-95-7948-01	476	0	T2	N0	M0	I	Female	42	-1.2618	Low	<=65
TCGA-95-8039-01	830	0	T1	N0	NA	I	Male	72	0.398315	High	>65

TCGA-95-8494-01	84	0	T2	N1	M0	II	Male	67	0.537503	High	>65
TCGA-95-A4VK-01	651	0	T2	N2	M0	III	Female	74	0.051667	High	>65
TCGA-95-A4VN-01	553	0	T2	N1	M0	II	Female	62	0.197099	High	<=65
TCGA-95-A4VP-01	605	0	T2	N2	M0	III	Female	66	-0.96948	Low	>65
TCGA-97-7546-01	1285	0	T1	N0	NA	I	Female	76	-0.84965	Low	>65
TCGA-97-7547-01	1965	0	T2	N0	NA	I	Female	67	-0.83604	Low	>65
TCGA-97-7552-01	1932	0	T2	N0	NA	I	Male	70	-1.04386	Low	>65
TCGA-97-7553-01	1870	0	T1	N0	NA	I	Female	58	-0.38678	Low	<=65
TCGA-97-7554-01	775	0	T2	N2	M0	III	Female	83	0.421105	High	>65
TCGA-97-7937-01	564	0	T2	N0	NA	I	Male	65	0.623374	High	<=65
TCGA-97-7938-01	18	1	T1	N0	NA	I	Female	76	-0.14364	Low	>65
TCGA-97-7941-01	484	0	T1	N0	NA	I	Female	72	-0.34307	Low	>65
TCGA-97-8171-01	568	0	T2	N2	M1	IV	Male	81	-0.99126	Low	>65
TCGA-97-8172-01	545	0	T2	N0	M0	I	Female	75	-0.85893	Low	>65

TCGA-97-8174-01	164	1	T2	N0	M0	II	Male	67	-1.89659	Low	>65
TCGA-97-8175-01	551	0	T2	N0	M0	I	Female	55	0.273041	High	<=65
TCGA-97-8176-01	468	1	T3	N1	M0	III	Male	63	0.968308	High	<=65
TCGA-97-8177-01	499	0	T2	N0	M0	I	Female	59	-0.84728	Low	<=65
TCGA-97-8179-01	435	0	T1	N0	M0	I	Male	72	0.113936	High	>65
TCGA-97-8547-01	657	0	T2	N2	NA	III	Female	78	0.007	High	>65
TCGA-97-8552-01	626	0	T1	N0	NA	I	Female	55	-1.57384	Low	<=65
TCGA-97-A4LX-01	614	0	T2	N0	M0	I	Male	81	-1.67861	Low	>65
TCGA-97-A4M0-01	652	0	T2	N0	M0	I	Female	60	-0.5737	Low	<=65
TCGA-97-A4M1-01	601	0	T1	N0	M0	I	Female	52	-1.86146	Low	<=65
TCGA-97-A4M2-01	624	0	T1	N0	M0	I	Male	66	-2.08707	Low	>65
TCGA-97-A4M3-01	540	0	T1	N0	M0	I	Female	69	0.575009	High	>65
TCGA-97-A4M5-01	634	0	T1	N0	M0	I	Male	83	-0.46457	Low	>65
TCGA-97-A4M6-01	568	0	T1	N0	M0	I	Female	45	-1.29242	Low	<=65

TCGA-97-A4M7-01	629	0	T1	N0	M0	I	Male	74	-1.37597	Low	>65
TCGA-99-7458-01	747	0	T4	N0	M0	III	Female	74	-0.48216	Low	>65
TCGA-99-8025-01	1060	0	T3	N2	M0	III	Female	72	0.385735	High	>65
TCGA-99-8028-01	1118	0	T1	N0	M0	I	Female	50	-0.05046	Low	<=65
TCGA-99-8032-01	44	0	T1	N0	M0	I	Male	61	-0.54323	Low	<=65
TCGA-99-8033-01	656	1	TX	NA	M1	IV	Female	74	1.877882	High	>65
TCGA-99-AA5R-01	658	0	T1	N0	M0	I	Female	70	-2.10252	Low	>65
TCGA-J2-8192-01	739	0	T2	N1	NA	II	Female	65	-1.26023	Low	<=65
TCGA-J2-8194-01	724	0	T3	N0	NA	II	Female	69	0.571166	High	>65
TCGA-J2-A4AD-01	550	1	T1	N0	NA	I	Female	61	0.211926	High	<=65
TCGA-J2-A4AE-01	1079	0	T1	N0	NA	I	Female	77	0.045001	High	>65
TCGA-J2-A4AG-01	988	0	T1	N0	NA	I	Female	66	-1.05083	Low	>65
TCGA-L4-A4E5-01	578	0	T1	N0	M0	I	Female	48	0.040401	High	<=65
TCGA-L4-A4E6-01	435	0	T1	N0	M0	I	Male	67	-2.06179	Low	>65

TCGA-L9-A443-01	193	1	T1	N0	NA	I	Female	63	0.042044	High	<=65
TCGA-L9-A444-01	307	0	T1	N0	NA	I	Female	60	-0.58998	Low	<=65
TCGA-L9-A50W-01	442	1	T1	N1	NA	II	Male	75	0.247846	High	>65
TCGA-L9-A5IP-01	58	1	T3	N2	M1	IV	Female	40	2.276526	High	<=65
TCGA-L9-A743-01	664	0	T2	N1	M0	II	Male	56	-0.09477	Low	<=65
TCGA-L9-A7SV-01	565	0	T2	N1	M0	II	Male	69	-1.68888	Low	>65
TCGA-L9-A8F4-01	476	0	T2	N0	NA	I	Female	64	-1.7259	Low	<=65
TCGA-MN-A4N1-01	827	0	T2	N1	M0	II	Male	60	0.206351	High	<=65
TCGA-MN-A4N4-01	1175	0	T1	N0	M0	I	Male	57	0.00233	High	<=65
TCGA-MN-A4N5-01	84	0	T1	N0	M0	I	Male	63	0.701473	High	<=65
TCGA-MP-A4SV-01	2620	1	T2	N0	M0	I	Male	67	-0.23383	Low	>65
TCGA-MP-A4SW-01	1778	1	T2	N1	M0	II	Male	53	-0.51924	Low	<=65
TCGA-MP-A4SY-01	1501	1	T2	N1	M0	II	Male	61	0.291668	High	<=65
TCGA-MP-A4T4-01	2617	1	T2	N1	M0	II	Female	68	-0.60227	Low	>65

TCGA-MP-A4T6-01	1790	1	T1	N2	NA	III	Female	76	-1.1589	Low	>65
TCGA-MP-A4T7-01	167	1	T2	N0	M1	IV	Female	75	1.8959	High	>65
TCGA-MP-A4T8-01	161	1	T2	N2	M0	III	Male	68	1.30631	High	>65
TCGA-MP-A4T9-01	1265	1	T2	N2	NA	III	Female	54	-0.38669	Low	<=65
TCGA-MP-A4TA-01	950	1	T1	N0	M0	I	Female	75	0.851689	High	>65
TCGA-MP-A4TC-01	74	1	T1	N2	M0	III	Male	77	0.263573	High	>65
TCGA-MP-A4TD-01	307	1	T2	N2	M0	III	Male	71	-0.39988	Low	>65
TCGA-MP-A4TE-01	896	1	T2	N0	NA	II	Male	56	0.378595	High	<=65
TCGA-MP-A4TF-01	336	1	T2	N0	M0	II	Female	58	2.821665	High	<=65
TCGA-MP-A4TH-01	741	0	T1	N0	M0	I	Female	70	-1.38644	Low	>65
TCGA-MP-A4TI-01	429	1	T2	N1	M0	II	Male	72	0.655642	High	>65
TCGA-MP-A4TJ-01	339	1	T1	N0	M0	I	Female	62	-0.65769	Low	<=65
TCGA-MP-A4TK-01	582	1	T2	N1	NA	II	Female	56	0.353832	High	<=65
TCGA-MP-A5C7-01	2248	0	T2	N0	M0	I	Female	76	-1.28672	Low	>65

TCGA-NJ-A4YF-01	2161	0	T1	N0	M0	I	Female	50	-0.0406	Low	<=65
TCGA-NJ-A4YG-01	2261	0	T2	N0	M0	I	Male	65	-0.2155	Low	<=65
TCGA-NJ-A4YI-01	4	1	T2	N2	M0	III	Female	87	-0.38246	Low	>65
TCGA-NJ-A4YP-01	50	0	T2	N0	M0	I	Male	52	1.370089	High	<=65
TCGA-NJ-A4YQ-01	1432	0	T1	N0	M0	I	Female	69	-0.32553	Low	>65
TCGA-NJ-A55A-01	15	0	T2	N0	M0	I	Female	76	-1.26386	Low	>65
TCGA-NJ-A55O-01	13	0	T1	N1	M0	II	Female	56	-0.64234	Low	<=65
TCGA-NJ-A55R-01	603	0	T1	N0	NA	I	Male	67	-1.23826	Low	>65
TCGA-NJ-A7XG-01	617	0	T4	N1	M0	III	Male	49	0.064398	High	<=65
TCGA-O1-A52J-01	1798	1	T1	N0	NA	I	Female	74	-0.81576	Low	>65
TCGA-S2-AA1A-01	513	0	T1	N0	M0	I	Female	68	-1.05492	Low	>65

Supplementary Table2: Clinical characteristics of each sample in GSE31210 cohort

Samples	Age	Gender	smoking_status	Stage	gene_alteration_status	OS.time	OS	RiskScore	type
GSM773540	55	female	Never-smoker	II	ALK-fusion +	437	dead	-2.88676	High
GSM773541	38	female	Ever-smoker	II	ALK-fusion +	743	alive	-2.71245	High
GSM773542	30	male	Ever-smoker	II	ALK-fusion +	2601	alive	-2.77904	High
GSM773543	64	female	Never-smoker	II	ALK-fusion +	1173	alive	-2.3935	High
GSM773544	63	female	Never-smoker	II	ALK-fusion +	2369	alive	-3.59549	LOW
GSM773545	60	female	Never-smoker	II	ALK-fusion +	1879	alive	-3.69871	LOW
GSM773546	68	female	Never-smoker	II	ALK-fusion +	606	alive	-3.09284	High
GSM773547	58	male	Ever-smoker	II	ALK-fusion +	300	dead	-3.41626	LOW
GSM773548	63	female	Never-smoker	IA	ALK-fusion +	1919	alive	-3.09952	High
GSM773549	56	female	Never-smoker	IA	ALK-fusion +	1835	alive	-3.01195	High
GSM773550	34	female	Ever-smoker	IA	ALK-fusion +	1828	alive	-2.69436	High
GSM773551	75	female	Never-smoker	II	KRAS mutation +	1841	dead	-3.48445	LOW
GSM773552	60	male	Ever-smoker	II	KRAS mutation +	639	dead	-2.35608	High

GSM773553	46	female	Never-smoker	IB	KRAS mutation +	1311	dead	-2.35266	High
GSM773554	60	female	Never-smoker	IB	KRAS mutation +	1098	alive	-2.30248	High
GSM773555	61	male	Ever-smoker	IA	KRAS mutation +	1189	alive	-2.41086	High
GSM773556	63	male	Ever-smoker	IA	KRAS mutation +	3863	alive	-2.47177	High
GSM773557	47	male	Ever-smoker	II	KRAS mutation +	1475	alive	-3.39698	LOW
GSM773558	59	male	Ever-smoker	II	KRAS mutation +	1856	alive	-3.39436	LOW
GSM773559	64	male	Ever-smoker	II	KRAS mutation +	3067	alive	-2.67737	High
GSM773560	60	male	Ever-smoker	II	KRAS mutation +	1142	alive	-2.84696	High
GSM773561	61	male	Never-smoker	IB	KRAS mutation +	1825	alive	-4.15098	LOW
GSM773562	53	female	Never-smoker	IB	KRAS mutation +	1610	alive	-3.47352	LOW
GSM773563	53	male	Ever-smoker	IB	KRAS mutation +	2014	alive	-2.85069	High
GSM773564	53	male	Ever-smoker	IB	KRAS mutation +	1826	alive	-2.96554	High
GSM773565	67	male	Never-smoker	IB	KRAS mutation +	2191	alive	-2.83277	High
GSM773566	69	female	Never-smoker	IB	KRAS mutation +	3342	alive	-3.0635	High
GSM773567	64	male	Ever-smoker	IA	KRAS mutation +	1328	alive	-3.22691	High

GSM773568	69	female	Never-smoker	IA	KRAS mutation +	2925	alive	-2.74531	High
GSM773569	50	female	Never-smoker	IA	KRAS mutation +	2880	alive	-3.43062	LOW
GSM773570	70	female	Never-smoker	IA	KRAS mutation +	1724	alive	-3.86754	LOW
GSM773571	57	male	Never-smoker	II	EGFR/KRAS/ALK -	2765	alive	-3.63839	LOW
GSM773572	58	male	Ever-smoker	II	EGFR/KRAS/ALK -	1529	alive	-3.34697	LOW
GSM773573	69	male	Ever-smoker	II	EGFR/KRAS/ALK -	221	dead	-2.35365	High
GSM773574	61	male	Ever-smoker	II	EGFR/KRAS/ALK -	546	dead	-3.52162	LOW
GSM773575	68	male	Ever-smoker	II	EGFR/KRAS/ALK -	346	dead	-2.22064	High
GSM773576	64	female	Never-smoker	II	EGFR/KRAS/ALK -	853	dead	-3.69891	LOW
GSM773577	69	female	Never-smoker	II	EGFR/KRAS/ALK -	2661	dead	-3.82444	LOW
GSM773578	66	female	Never-smoker	II	EGFR/KRAS/ALK -	817	alive	-3.1278	High
GSM773579	68	male	Ever-smoker	II	EGFR/KRAS/ALK -	425	dead	-3.29977	LOW
GSM773580	69	male	Ever-smoker	II	EGFR/KRAS/ALK -	1281	alive	-4.17525	LOW
GSM773581	64	male	Ever-smoker	IB	EGFR/KRAS/ALK -	1044	alive	-2.40488	High
GSM773582	64	male	Ever-smoker	IB	EGFR/KRAS/ALK -	565	alive	-1.86749	High

GSM773583	66	male	Ever-smoker	IB	EGFR/KRAS/ALK -	1059	dead	-3.29226	LOW
GSM773584	64	male	Ever-smoker	IB	EGFR/KRAS/ALK -	1625	dead	-3.25273	High
GSM773585	68	female	Never-smoker	IB	EGFR/KRAS/ALK -	1695	dead	-3.40673	LOW
GSM773586	68	male	Ever-smoker	IB	EGFR/KRAS/ALK -	2976	alive	-2.96377	High
GSM773587	63	male	Ever-smoker	IB	EGFR/KRAS/ALK -	1143	dead	-2.92936	High
GSM773588	58	female	Never-smoker	IB	EGFR/KRAS/ALK -	1682	alive	-3.01395	High
GSM773589	71	male	Ever-smoker	IB	EGFR/KRAS/ALK -	1242	dead	-3.18051	High
GSM773590	52	female	Never-smoker	IB	EGFR/KRAS/ALK -	2252	alive	-3.41011	LOW
GSM773591	66	male	Ever-smoker	IA	EGFR/KRAS/ALK -	943	dead	-3.06168	High
GSM773592	66	female	Never-smoker	IA	EGFR/KRAS/ALK -	1346	dead	-2.9093	High
GSM773593	47	female	Never-smoker	IA	EGFR/KRAS/ALK -	1482	dead	-2.8382	High
GSM773594	64	male	Never-smoker	IA	EGFR/KRAS/ALK -	2386	alive	-3.90464	LOW
GSM773595	54	male	Never-smoker	IA	EGFR/KRAS/ALK -	1560	alive	-3.0647	High
GSM773596	67	female	Never-smoker	IA	EGFR/KRAS/ALK -	1001	dead	-3.39206	LOW
GSM773597	54	female	Never-smoker	II	EGFR/KRAS/ALK -	259	dead	-2.80722	High

GSM773598	65	male	Ever-smoker	II	EGFR/KRAS/ALK -	2289	alive	-1.85386	High
GSM773599	49	female	Ever-smoker	II	EGFR/KRAS/ALK -	1916	alive	-2.56212	High
GSM773600	62	male	Ever-smoker	II	EGFR/KRAS/ALK -	2048	alive	-3.53189	LOW
GSM773601	61	male	Ever-smoker	II	EGFR/KRAS/ALK -	1475	alive	-2.62482	High
GSM773602	57	male	Ever-smoker	II	EGFR/KRAS/ALK -	1539	alive	-3.93331	LOW
GSM773603	60	male	Ever-smoker	II	EGFR/KRAS/ALK -	636	alive	-2.9892	High
GSM773604	46	male	Ever-smoker	II	EGFR/KRAS/ALK -	1275	alive	-2.60197	High
GSM773605	52	male	Ever-smoker	II	EGFR/KRAS/ALK -	2005	alive	-4.07769	LOW
GSM773606	54	female	Never-smoker	II	EGFR/KRAS/ALK -	1965	alive	-3.40956	LOW
GSM773607	58	female	Never-smoker	IB	EGFR/KRAS/ALK -	2425	alive	-3.61436	LOW
GSM773608	64	female	Never-smoker	IB	EGFR/KRAS/ALK -	1566	alive	-4.41711	LOW
GSM773609	65	female	Never-smoker	IB	EGFR/KRAS/ALK -	990	alive	-3.61445	LOW
GSM773610	52	male	Ever-smoker	IB	EGFR/KRAS/ALK -	729	alive	-3.35254	LOW
GSM773611	61	male	Never-smoker	IB	EGFR/KRAS/ALK -	2354	alive	-3.16464	High
GSM773612	61	male	Ever-smoker	IB	EGFR/KRAS/ALK -	818	alive	-3.91683	LOW

GSM773613	62	female	Ever-smoker	IB	EGFR/KRAS/ALK -	2009	alive	-3.29503	LOW
GSM773614	47	female	Ever-smoker	IB	EGFR/KRAS/ALK -	2549	alive	-2.38063	High
GSM773615	64	male	Ever-smoker	IB	EGFR/KRAS/ALK -	3411	alive	-2.80613	High
GSM773616	50	male	Ever-smoker	IB	EGFR/KRAS/ALK -	1806	alive	-2.53839	High
GSM773617	66	female	Never-smoker	IA	EGFR/KRAS/ALK -	1937	alive	-2.84927	High
GSM773618	56	male	Never-smoker	IA	EGFR/KRAS/ALK -	1520	alive	-3.06269	High
GSM773619	76	male	Ever-smoker	IA	EGFR/KRAS/ALK -	1375	alive	-3.34773	LOW
GSM773620	56	female	Never-smoker	IA	EGFR/KRAS/ALK -	1756	alive	-3.36473	LOW
GSM773621	53	male	Ever-smoker	IA	EGFR/KRAS/ALK -	1597	alive	-2.31802	High
GSM773622	57	male	Never-smoker	IA	EGFR/KRAS/ALK -	1714	alive	-3.47732	LOW
GSM773623	61	female	Never-smoker	IA	EGFR/KRAS/ALK -	1637	alive	-3.19174	High
GSM773624	66	female	Never-smoker	IA	EGFR/KRAS/ALK -	1653	alive	-3.74178	LOW
GSM773625	66	male	Ever-smoker	IA	EGFR/KRAS/ALK -	1196	alive	-3.47193	LOW
GSM773626	57	male	Never-smoker	IA	EGFR/KRAS/ALK -	1702	alive	-3.46847	LOW
GSM773627	61	female	Never-smoker	IA	EGFR/KRAS/ALK -	2050	alive	-3.2192	High

GSM773628	63	male	Ever-smoker	IA	EGFR/KRAS/ALK -	1256	alive	-3.61016	LOW
GSM773629	60	male	Ever-smoker	IA	EGFR/KRAS/ALK -	818	alive	-4.15717	LOW
GSM773630	59	male	Ever-smoker	IA	EGFR/KRAS/ALK -	1154	alive	-2.53748	High
GSM773631	55	female	Never-smoker	IA	EGFR/KRAS/ALK -	3058	alive	-3.24152	High
GSM773632	52	male	Ever-smoker	IA	EGFR/KRAS/ALK -	1941	alive	-3.21815	High
GSM773633	68	female	Never-smoker	IA	EGFR/KRAS/ALK -	1596	alive	-2.58928	High
GSM773634	55	male	Ever-smoker	IA	EGFR/KRAS/ALK -	2450	alive	-3.45731	LOW
GSM773635	64	female	Never-smoker	IA	EGFR/KRAS/ALK -	1881	alive	-4.15736	LOW
GSM773636	51	male	Ever-smoker	IA	EGFR/KRAS/ALK -	1637	alive	-3.55041	LOW
GSM773637	55	female	Never-smoker	IA	EGFR/KRAS/ALK -	2034	alive	-2.58035	High
GSM773638	65	female	Never-smoker	IA	EGFR/KRAS/ALK -	2134	alive	-4.09139	LOW
GSM773639	60	female	Never-smoker	II	EGFR mutation +	779	dead	-1.76849	High
GSM773640	58	male	Ever-smoker	II	EGFR mutation +	540	dead	-2.53376	High
GSM773641	68	male	Ever-smoker	II	EGFR mutation +	1317	alive	-2.22769	High

GSM773642	65	female	Never-smoker	II	EGFR mutation +	1980	alive	-2.85974	High
GSM773643	66	male	Ever-smoker	II	EGFR mutation +	702	dead	-3.21357	High
GSM773644	66	male	Ever-smoker	II	EGFR mutation +	564	dead	-2.93001	High
GSM773645	62	male	Ever-smoker	II	EGFR mutation +	788	dead	-3.05015	High
GSM773646	58	female	Ever-smoker	II	EGFR mutation +	1463	alive	-3.24131	High
GSM773647	49	male	Ever-smoker	II	EGFR mutation +	1345	dead	-3.31128	LOW
GSM773648	61	male	Ever-smoker	II	EGFR mutation +	832	alive	-3.09101	High
GSM773649	59	female	Never-smoker	II	EGFR mutation +	2687	alive	-3.63101	LOW
GSM773650	53	female	Never-smoker	II	EGFR mutation +	1122	dead	-2.79363	High
GSM773651	64	male	Ever-smoker	II	EGFR mutation +	2668	alive	-3.29181	LOW
GSM773652	67	female	Never-smoker	IB	EGFR mutation +	1936	alive	-3.42873	LOW
GSM773653	69	male	Ever-smoker	IB	EGFR mutation +	871	alive	-3.37728	LOW
GSM773654	61	female	Never-smoker	IB	EGFR mutation +	2671	alive	-2.63195	High
GSM773655	64	female	Ever-smoker	IB	EGFR mutation +	1482	alive	-2.36169	High

GSM773656	53	female	Never-smoker	IB	EGFR mutation +	1128	alive	-3.75004	LOW
GSM773657	71	female	Never-smoker	IB	EGFR mutation +	1042	dead	-3.68198	LOW
GSM773658	52	male	Never-smoker	IB	EGFR mutation +	1229	dead	-2.86002	High
GSM773659	52	female	Never-smoker	IB	EGFR mutation +	2263	dead	-2.8682	High
GSM773660	54	female	Never-smoker	IA	EGFR mutation +	1770	alive	-2.95151	High
GSM773661	49	male	Ever-smoker	IA	EGFR mutation +	1433	dead	-2.40426	High
GSM773662	68	female	Never-smoker	IA	EGFR mutation +	1823	alive	-3.36624	LOW
GSM773663	62	female	Never-smoker	IA	EGFR mutation +	1988	alive	-2.31938	High
GSM773664	63	female	Ever-smoker	IA	EGFR mutation +	1434	alive	-3.33841	LOW
GSM773665	63	male	Ever-smoker	IA	EGFR mutation +	630	dead	-3.16689	High
GSM773666	62	female	Never-smoker	IA	EGFR mutation +	1537	alive	-3.43598	LOW
GSM773667	57	female	Ever-smoker	IA	EGFR mutation +	1429	dead	-3.10108	High
GSM773668	39	female	Ever-smoker	IA	EGFR mutation +	1038	dead	-3.25822	High
GSM773669	55	female	Never-smoker	II	EGFR mutation +	2486	alive	-3.2399	High
GSM773670	47	male	Ever-smoker	II	EGFR mutation +	740	alive	-3.58661	LOW

GSM773671	64	female	Never-smoker	II	EGFR mutation +	1042	alive	-4.27946	LOW
GSM773672	60	female	Ever-smoker	II	EGFR mutation +	1267	alive	-3.88932	LOW
GSM773673	48	female	Never-smoker	II	EGFR mutation +	1017	alive	-2.51002	High
GSM773674	60	male	Ever-smoker	II	EGFR mutation +	1041	alive	-2.73068	High
GSM773675	52	male	Ever-smoker	II	EGFR mutation +	1859	alive	-2.83508	High
GSM773676	48	female	Never-smoker	II	EGFR mutation +	1717	alive	-2.7084	High
GSM773677	60	female	Never-smoker	II	EGFR mutation +	1494	alive	-3.49917	LOW
GSM773678	49	male	Ever-smoker	II	EGFR mutation +	754	alive	-3.80325	LOW
GSM773679	63	male	Ever-smoker	II	EGFR mutation +	2364	alive	-3.8449	LOW
GSM773680	68	male	Ever-smoker	IB	EGFR mutation +	1733	alive	-2.88818	High
GSM773681	58	female	Never-smoker	IB	EGFR mutation +	1941	alive	-3.88986	LOW
GSM773682	68	male	Ever-smoker	IB	EGFR mutation +	774	alive	-3.28046	LOW
GSM773683	64	female	Never-smoker	IB	EGFR mutation +	2913	alive	-2.98609	High
GSM773684	58	male	Ever-smoker	IB	EGFR mutation +	1803	alive	-3.43997	LOW
GSM773685	62	male	Ever-smoker	IB	EGFR mutation +	1912	alive	-2.91633	High

GSM773686	66	female	Never-smoker	IB	EGFR mutation +	1481	alive	-4.10403	LOW
GSM773687	68	female	Ever-smoker	IB	EGFR mutation +	1231	alive	-2.98283	High
GSM773688	68	male	Never-smoker	IB	EGFR mutation +	1964	alive	-3.20232	High
GSM773689	72	male	Ever-smoker	IB	EGFR mutation +	3352	alive	-3.85751	LOW
GSM773690	62	male	Ever-smoker	IB	EGFR mutation +	2158	alive	-3.32962	LOW
GSM773691	67	female	Never-smoker	IB	EGFR mutation +	3466	alive	-3.52087	LOW
GSM773692	52	male	Never-smoker	IB	EGFR mutation +	3263	alive	-3.05798	High
GSM773693	67	female	Never-smoker	IB	EGFR mutation +	1823	alive	-3.37764	LOW
GSM773694	59	female	Never-smoker	IB	EGFR mutation +	1856	alive	-3.51751	LOW
GSM773695	70	female	Never-smoker	IB	EGFR mutation +	2449	alive	-3.78994	LOW
GSM773696	59	male	Ever-smoker	IB	EGFR mutation +	1859	alive	-3.75028	LOW
GSM773697	56	male	Ever-smoker	IB	EGFR mutation +	1998	alive	-3.01351	High
GSM773698	69	female	Ever-smoker	IA	EGFR mutation +	1852	alive	-3.51071	LOW
GSM773699	61	female	Ever-smoker	IA	EGFR mutation +	1894	alive	-3.00621	High

GSM773700	54	female	Never-smoker	IA	EGFR mutation +	1861	alive	-3.52669	LOW
GSM773701	64	female	Never-smoker	IA	EGFR mutation +	1669	alive	-4.14086	LOW
GSM773702	45	female	Never-smoker	IA	EGFR mutation +	3189	alive	-2.5954	High
GSM773703	55	female	Never-smoker	IA	EGFR mutation +	1822	alive	-3.81172	LOW
GSM773704	61	female	Never-smoker	IA	EGFR mutation +	1809	alive	-4.14603	LOW
GSM773705	65	female	Never-smoker	IA	EGFR mutation +	2552	alive	-3.35278	LOW
GSM773706	65	male	Never-smoker	IA	EGFR mutation +	2968	alive	-3.23607	High
GSM773707	47	male	Ever-smoker	IA	EGFR mutation +	3066	alive	-2.78787	High
GSM773708	60	female	Never-smoker	IA	EGFR mutation +	1412	alive	-2.77609	High
GSM773709	55	male	Ever-smoker	IA	EGFR mutation +	1317	alive	-5.1171	LOW
GSM773710	61	female	Never-smoker	IA	EGFR mutation +	1180	alive	-3.26795	High
GSM773711	67	male	Ever-smoker	IA	EGFR mutation +	1688	alive	-3.36529	LOW
GSM773712	69	male	Ever-smoker	IA	EGFR mutation +	1417	alive	-3.97339	LOW
GSM773713	65	female	Never-smoker	IA	EGFR mutation +	1426	alive	-3.01317	High
GSM773714	65	male	Ever-smoker	IA	EGFR mutation +	1674	alive	-2.60897	High

GSM773715	56	female	Never-smoker	IA	EGFR mutation +	1848	alive	-2.41155	High
GSM773716	65	female	Ever-smoker	IA	EGFR mutation +	1856	alive	-3.63701	LOW
GSM773717	56	female	Never-smoker	IA	EGFR mutation +	1780	alive	-2.92735	High
GSM773718	59	male	Never-smoker	IA	EGFR mutation +	2084	alive	-3.84648	LOW
GSM773719	56	male	Ever-smoker	IA	EGFR mutation +	1879	alive	-3.44185	LOW
GSM773720	63	female	Ever-smoker	IA	EGFR mutation +	1114	alive	-2.67042	High
GSM773721	49	male	Ever-smoker	IA	EGFR mutation +	2806	alive	-3.85458	LOW
GSM773722	58	female	Never-smoker	IA	EGFR mutation +	1663	alive	-2.88731	High
GSM773723	62	male	Ever-smoker	IA	EGFR mutation +	1726	alive	-4.24918	LOW
GSM773724	68	female	Never-smoker	IA	EGFR mutation +	1629	alive	-3.40455	LOW
GSM773725	66	female	Never-smoker	IA	EGFR mutation +	2179	alive	-3.7687	LOW
GSM773726	57	female	Ever-smoker	IA	EGFR mutation +	1917	alive	-3.86616	LOW
GSM773727	57	female	Never-smoker	IA	EGFR mutation +	1446	alive	-2.73101	High
GSM773728	52	female	Ever-smoker	IA	EGFR mutation +	1162	alive	-3.32866	LOW
GSM773729	65	female	Never-smoker	IA	EGFR mutation +	1231	alive	-2.78057	High

GSM773730	65	male	Ever-smoker	IA	EGFR mutation +	941	alive	-2.66203	High
GSM773731	50	female	Never-smoker	IA	EGFR mutation +	1623	alive	-4.08103	LOW
GSM773732	53	female	Never-smoker	IA	EGFR mutation +	1855	alive	-3.27619	High
GSM773733	53	female	Never-smoker	IA	EGFR mutation +	1404	alive	-3.09662	High
GSM773734	66	female	Ever-smoker	IA	EGFR mutation +	2221	alive	-4.43447	LOW
GSM773735	62	female	Never-smoker	IA	EGFR mutation +	849	alive	-3.74179	LOW
GSM773736	66	female	Never-smoker	IA	EGFR mutation +	831	alive	-3.42726	LOW
GSM773737	35	male	Ever-smoker	IA	EGFR mutation +	3219	alive	-3.60665	LOW
GSM773738	59	female	Never-smoker	IA	EGFR mutation +	2585	alive	-2.52814	High
GSM773739	67	female	Never-smoker	IA	EGFR mutation +	2442	alive	-3.78197	LOW
GSM773740	56	female	Ever-smoker	IA	EGFR mutation +	1910	alive	-4.14353	LOW
GSM773741	62	female	Never-smoker	IA	EGFR mutation +	1834	alive	-3.32139	LOW
GSM773742	46	female	Never-smoker	IA	EGFR mutation +	2918	alive	-2.91884	High
GSM773743	67	female	Never-smoker	IA	EGFR mutation +	2285	alive	-3.84538	LOW

GSM773744	62	male	Ever-smoker	IA	EGFR mutation +	2149	alive	-2.87557	High
GSM773745	63	male	Ever-smoker	IA	EGFR mutation +	2561	alive	-3.19839	High
GSM773746	52	female	Ever-smoker	IA	EGFR mutation +	1947	alive	-3.35113	LOW
GSM773747	60	female	Never-smoker	IA	EGFR mutation +	1841	alive	-3.01942	High
GSM773748	47	female	Never-smoker	IA	EGFR mutation +	1971	alive	-4.06973	LOW
GSM773749	56	male	Ever-smoker	IA	EGFR mutation +	2409	alive	-3.5262	LOW
GSM773750	56	female	Never-smoker	IA	EGFR mutation +	1624	alive	-3.53298	LOW
GSM773751	63	female	Ever-smoker	IA	EGFR mutation +	2430	alive	-3.51465	LOW
GSM773752	59	male	Ever-smoker	IA	EGFR mutation +	1856	alive	-3.36334	LOW
GSM773753	62	female	Never-smoker	IA	EGFR mutation +	1516	alive	-3.03433	High
GSM773754	50	male	Ever-smoker	IA	EGFR mutation +	1819	alive	-3.70559	LOW
GSM773755	55	male	Never-smoker	IA	EGFR mutation +	2413	alive	-3.28337	LOW
GSM773756	54	female	Ever-smoker	IA	EGFR mutation +	1849	alive	-3.62379	LOW
GSM773757	65	male	Never-smoker	IA	EGFR mutation +	2181	alive	-3.4696	LOW
GSM773758	66	male	Ever-smoker	IA	EGFR mutation +	2168	alive	-3.23017	High

GSM773759	62	female	Never-smoker	IA	EGFR mutation +	2154	alive	-3.63522	LOW
GSM773760	64	female	Never-smoker	IA	EGFR mutation +	2073	alive	-3.80235	LOW
GSM773761	64	female	Never-smoker	IA	EGFR mutation +	1994	alive	-3.59452	LOW
GSM773762	61	male	Never-smoker	IA	EGFR mutation +	2137	alive	-2.94012	High
GSM773763	58	female	Never-smoker	IA	EGFR mutation +	1998	alive	-3.12419	High
GSM773764	49	male	Ever-smoker	IA	EGFR mutation +	1908	alive	-3.56415	LOW
GSM773765	58	male	Ever-smoker	IA	EGFR mutation +	1660	alive	-4.24844	LOW
